# Statistics of the Compression Ratio of a Variable-to-Variable Code: Exact Moments and Asymptotic Behavior

**DOI:** 10.3390/e28080898

**Published:** 2026-08-10

**Authors:** Neri Merhav

**Affiliations:** The Viterbi Faculty of Electrical and Computer Engineering, Technion—Israel Institute of Technology, Technion City, Haifa 3200003, Israel; merhav@technion.ac.il

**Keywords:** variable-to-variable codes, source coding, compression ratio, finite-length analysis, exact moments, Markov sources, Edgeworth expansion, large deviations

## Abstract

A variable-to-variable (V2V) length code parses a source sequence into phrases of variable length and maps each phrase to a binary codeword of, generally, a different random length. After encoding *n* phrases, the realized compression ratio $R_{n}=\Lambda_{n}/\Sigma_{n}$—total codeword length over total source-symbol count—is the finite-sample counterpart of the code’s asymptotic rate ρ, to which it converges only as n→∞. This paper first derives exact formulas for all integer moments of $R_{n}$ for a given discrete memoryless source (DMS). Specifically, we obtain a closed-form formula for every moment $E\{R_{n}^{k}\}$ as a one-dimensional integral involving only single-phrase moment generating functions of the pair (L,l)—the phrase length, in source symbols, and codeword length, in bits. From these moments we derive an Edgeworth approximation to the cumulative distribution function (CDF) of $R_{n}$ that is substantially more accurate than the central limit theorem (CLT) approximation. Using the Laplace method of integration, we also derive explicit closed-form formulas for the bias constant $C=\lim_{n\to \infty }n\left(E\left\{R_{n}\right\}-\rho \right)$ and for the variance constant $\lim_{n\to \infty }n\cdot \mathrm{Var}\left\{R_{n}\right\}$. The analysis extends to Markov sources via state-indexed matrices with a redundancy formula obtained in closed form. On the coding-theoretic side, we cast V2V length codes as finite-state encoders and apply a generalized Kraft inequality for a compression-rate lower bound, and give a structural decomposition of the bias coefficient that separates cleanly across variable-to-fixed (V2F) length codes, fixed-to-variable (F2V) length codes, and V2V length codes. Applied to the Khodak code of Bugeaud, Drmota, and Szpankowski, this decomposition shows that its improved performance is reflected in its smaller bias constant.

## 1. Introduction

### 1.1. Objectives and Motivation

A variable-to-variable (V2V) length source code is the most general member of the family of lossless codes considered here. It parses the source into phrases of variable length and encodes each phrase with a binary codeword of variable length, combining the structural advantages of variable-to-fixed (V2F) length coding—which adapts the parsing to source statistics—with those of fixed-to-variable (F2V) length coding—which adapts codeword lengths to symbol probabilities.

The standard performance measure for a V2V length code is its asymptotic compression rate, ρ=E{l}/E{L}, the ratio of expected codeword length to expected phrase length. Let $\Lambda_{n}$ and $\Sigma_{n}$ denote, respectively, the total codeword length (in bits) and the total source-symbol count after *n* encoded phrases. By the strong law of large numbers ($\Lambda_{n}/n\to E\left\{l\right\}$ and $\Sigma_{n}/n\to E\left\{L\right\}$ almost surely) and the continuous mapping theorem [1], the realized compression ratio $R_{n}=\Lambda_{n}/\Sigma_{n}$ converges to ρ almost surely as n→∞. This asymptotic rate, however, answers neither how quickly convergence occurs nor how large the fluctuations around ρ are at any finite number of encoded phrases *n*. Estimating or bounding a code’s realized performance at finite *n* is important in its own right—for instance, when comparing candidate codes before the asymptotic regime is reached—and is closely related to, though distinct from, the classical question of buffer overflow, where the object of interest is the joint behavior of $\Lambda_{n}$ and $\Sigma_{n}$ rather than their ratio alone (see Related Work below). Both questions are in fact governed by the same underlying object: the single-phrase joint moment generating function $\mu \left(\theta ,t\right):=E\left\{e^{\theta l(Y)-tL(Y)}\right\}$, since $E\left\{e^{\theta \Lambda_{n}-t\Sigma_{n}}\right\}=\mu^{n}\left(\theta ,t\right)$ for an i.i.d. source carries the full information about the joint distribution of $\left(\Lambda_{n},\Sigma_{n}\right)$. The exact moment formulas derived here use only the single-phrase quantities $\mu_{j}\left(t\right)=\partial_{\theta }^{j}{\mu \left(\theta ,t\right)|}_{\theta =0}$ (Section 2), the θ-derivatives of μ(θ,t) at the origin—sufficient for the moments of the ratio $R_{n}$ itself but not for joint tail probabilities such as buffer overflow; extending the present approach to that joint, two-parameter object is an interesting direction left for future work.

We address these questions by deriving exact formulas for all integer moments of $R_{n}$, for a given V2V length code and a memoryless source. This is fundamentally an analysis of a *given* code’s finite-sample behavior, not a code-design method; the joint design of the parsing tree and codeword lengths for a target *n* remains open (see Section 2.5). Our main result is an exact formula for $E\{R_{n}^{k}\}$ for every positive integer *k* as a single one-dimensional integral, requiring only the single-phrase quantities $\mu_{j}\left(t\right)$ introduced above. The underlying tool is an integral representation of $1/\Sigma_{n}$ as well as its *k*-th power as a Laplace transform, which enables turning a ratio of sums into a one-dimensional integral of simple single-phrase quantities. The novelty is in the systematic extension to *every* integer moment via the set-partition combinatorics of Theorem 4; the fact that the resulting formulas are exact at every finite *n* rather than asymptotic; and the further extension to Markov sources (Section 5), which replaces scalar moment generating functions by matrix-valued ones and requires a corresponding eigenvalue-perturbation argument with no scalar analogue. From these moments we derive closed-form asymptotic formulas for the bias constant $\lim_{n\to \infty }n\left(E\left\{R_{n}\right\}-\rho \right)$ and the variance constant $\lim_{n\to \infty }n\mathrm{Var}\left\{R_{n}\right\}$, and we construct an Edgeworth approximation to the CDF of $R_{n}$ that is substantially more accurate than the central limit theorem (CLT). Before turning to this analysis, Section 2 records some necessary background, including a compression-rate lower bound obtained by observing that a V2V length code is an instance of the finite-state encoders covered by the generalized Kraft inequality of [2]; this observation sets the stage for the moment analysis but is logically independent of it. Unlike the classical route to such a bound—concatenating many phrases and invoking a law-of-large-numbers argument to identify the limiting rate—the generalized-Kraft-inequality bound is a direct, purely algebraic consequence of a single spectral-radius inequality, with no block-length limit theorem involved.

### 1.2. Related Work

The V2F and V2V length coding literature is relatively sparse. Existing work, surveyed in [3], focuses on the average redundancy of specific named codes (Huffman, Tunstall, Khodak, Boncelet) for known sources, using analytic combinatorics and Mellin-transform methods; Savari and Szpankowski [4] give an early analysis specifically of V2V length codes along these lines. The moments of $R_{n}$ at finite *n*, as distinct from the asymptotic rate ρ, have not been addressed.

*V2F Length Coding* Tunstall’s algorithm [5] constructs an optimal uniquely parsable V2F dictionary by a simple greedy procedure. Savari and Gallager [6] established the asymptotic redundancy of Tunstall codes using renewal theory (modeling self-information as a regenerative process) and Markov reward machinery; their analysis brings in second moments of inter-renewal times, but only as a correction to the precision of an asymptotic mean formula, not as a variance target. Drmota, Reznik, and Szpankowski [7] established a CLT and a variance formula for the V2F phrase length *L*, via renewal theory and Mellin transforms, as the dictionary size M→∞. Both bodies of work address the phrase length *L* alone, under a fixed-length codeword assignment, as a function of a growing dictionary; our asymptotic variable is the number of phrases *n* processed by a fixed code, and our object is the ratio $R_{n}=\Lambda_{n}/\Sigma_{n}$ rather than *L*.
*Plurally Parsable Dictionaries* Savari [8,9] showed that relaxing unique parsability can outperform the Tunstall dictionary of the same size for highly predictable sources, with exact results for specific binary-source families but no general algorithm.
*Delay and Redundancy* Shayevitz, Meron, Feder, and Zamir [10] characterize the redundancy–delay trade-off for V2V and related code families. Their object is the expected per-symbol code length as a function of a worst-case delay constraint, not the moments of $R_{n}$ as the number of phrases grows.
*Buffer Overflow Analysis* A separate line of work studies the joint behavior of $\Lambda_{n}$ and $\Sigma_{n}$ directly, in order to bound the probability that an encoder’s output buffer overflows: Jelinek [11] derives the overflow exponent for fixed-rate discrete memoryless sources encoded with variable-length codewords; Wyner [12] extends this to arbitrary bounded input–output distributions; Humblet [13] generalizes Huffman coding itself to directly minimize overflow probability rather than expected length; and Merhav [14] studies universal coding schemes that minimize the probability of codeword-length overflow when the source statistics are unknown. This is a genuinely different question from the one addressed here: buffer overflow is governed by the joint tail behavior of $\left(\Lambda_{n},\Sigma_{n}\right)$ exceeding a threshold, whereas the present paper characterizes the distribution of the *ratio* $R_{n}=\Lambda_{n}/\Sigma_{n}$ itself; the two are related but distinct, as discussed further in Section 1.1.
*Codeword-Length Distributions* Courtade and Verdú [15] study the cumulant generating function (CGF) and Gaussian approximation of the codeword length of an optimal F2V length code on an *n*-symbol block, tracing back to Strassen’s foundational second-order asymptotic analysis [16] and complementing Kontoyiannis’s earlier second-order noiseless source coding theorems [17] (which also covers Markov sources) and the non-asymptotic refinements of Kontoyiannis and Verdú [18]. This is a single i.i.d. sum, not a ratio of two correlated sums with a random denominator; the ratio structure is specific to V2V and V2F coding and is the source of the combinatorial complexity addressed in the present paper.
*Generalized Kraft Inequality* Merhav [2] establishes a generalized Kraft inequality for finite-state encoders, strictly improving the earlier result of Ziv and Lempel [19]. We use this to derive an explicit lower bound on the V2V compression ratio in terms of the parsing tree structure.

## 2. System Model and Background on V2V Codes

### 2.1. Source Model and Notation

We consider a discrete memoryless source (DMS) $X_{1},X_{2},\ldots$, where each symbol $X_{i}$ (*i*—positive integer) is a random variable taking values in a finite alphabet X of cardinality α; specific realizations are denoted by $x_{1},x_{2},\ldots$. Each symbol is drawn according to the source distribution P={P(x),x∈X}. The single-letter entropy is

(1) $$H\left(X\right)=-\sum_{x\in X}P\left(x\right)\log_{2}P\left(x\right).$$

A V2V length code is specified by two components:

(1) A *source dictionary* D: A prefix-free set of variable-length source strings, represented by the leaves of a complete α-ary tree. The size of the dictionary is |D|=M, and a generic member of D is denoted by *y*. A source string $x_{1},x_{2},\ldots$ is parsed by the dictionary parser into a succession of phrases $y_{1},y_{2},\ldots$; the corresponding random variables $Y_{1},Y_{2},\ldots$, induced by the source *P*, form another DMS with alphabet D (of size *M*) and phrase probabilities {Q(y),y∈D}, where each Q(y) is given by the product of the letter probabilities that make up the phrase *y*. Let L(y), y∈D, denote the length of phrase *y*, in source symbols. We denote by $H\left(Y\right)\overset{\Delta }{=}-\sum_{y\in D}Q\left(y\right)\log_{2}Q\left(y\right)$ the phrase entropy.
(2) A variable-length uniquely decodable (UD) code, mapping D into a set of variable-length binary strings. Let l(y) denote the length, in bits, of the codeword assigned to y∈D.

We index the successive phrases produced by the parser by i=1,2,…; the *i*-th phrase is $Y_{i}$, of length $L(Y_{i})$ source symbols, encoded by a codeword of length $l(Y_{i})$ bits. For a DMS, the pairs ${\left(L\left(Y_{i}\right),l\left(Y_{i}\right)\right)}_{i\geq 1}$ are independent and identically distributed; we write *Y* for a generic random phrase with distribution *Q*, so that L(Y) and l(Y) denote its length and its codeword length, respectively. When it causes no ambiguity, we abbreviate L(Y) and l(Y) by *L* and *ℓ*; the explicit argument *Y* is retained whenever a formula defines a new named quantity as an expectation. The following single-phrase quantity appears throughout:

(2) $$\mu_{j}\left(t\right)\overset{\Delta }{=}E\left\{{\left[l\left(Y\right)\right]}^{j}e^{-tL(Y)}\right\},\;j=0,1,2,\ldots ,\;t\geq 0.$$

Note that $\mu_{0}\left(t\right)=E\left\{e^{-tL(Y)}\right\}$ is the MGF of −L(Y). We define the mean phrase length and mean codeword length by

(3) $$\bar{L}\overset{\Delta }{=}E\left\{L\left(Y\right)\right\}=\sum_{y\in D}Q\left(y\right)L\left(y\right),\;\bar{l}\overset{\Delta }{=}E\left\{l\left(Y\right)\right\}=\sum_{y\in D}Q\left(y\right)l\left(y\right).$$

The asymptotic compression rate of the code is defined as

(4) $$\rho \overset{\Delta }{=}\frac{\bar{l}}{\bar{L}}=-\frac{\mu_{1}\left(0\right)}{\mu_{0}^{\prime }\left(0\right)},$$

where $\mu_{0}^{\prime }\left(\cdot \right)$ denotes the derivative of $\mu_{0}\left(\cdot \right)$. For any two random variables U,V appearing below, we write $\mathrm{Var}\left\{U\right\}\overset{\Delta }{=}E\left\{U^{2}\right\}-{\left(E\left\{U\right\}\right)}^{2}$ and $\mathrm{Cov}\left\{U,V\right\}\overset{\Delta }{=}E\left\{UV\right\}-E\left\{U\right\}E\left\{V\right\}$ for their variance and covariance, respectively.

After encoding *n* phrases, the total code length is $\Lambda_{n}\overset{\Delta }{=}\sum_{i=1}^{n}l\left(Y_{i}\right)$ (in bits) and the total number of source symbols consumed is $\Sigma_{n}\overset{\Delta }{=}\sum_{i=1}^{n}L\left(Y_{i}\right)$. The *realized compression ratio* over *n* phrases is

(5) $$R_{n}\overset{\Delta }{=}\frac{\Lambda_{n}}{\Sigma_{n}}=\frac{\sum_{i=1}^{n}l\left(Y_{i}\right)}{\sum_{i=1}^{n}L\left(Y_{i}\right)}\;\;\frac{\mathrm{bits}}{\mathrm{symbol}}.$$

By the strong law of large numbers, $\Lambda_{n}/n\to \bar{l}$ and $\Sigma_{n}/n\to \bar{L}$ almost surely (a.s.), and so, by the continuous mapping theorem, $R_{n}\to \rho$ a.s. as n→∞.

### 2.2. Structure and Parsing

The dictionary D of a V2V length code is represented by a full α-ary rooted tree, i.e., every internal node has α children, one per each possible source symbol; the *M* leaves correspond to the *M* phrases y∈D. The parsing rule is as follows: start at the root, follow the edge labeled by each successive source symbol until reaching a leaf. The leaf reached identifies the current phrase *y*; the encoder emits the binary codeword assigned to that leaf and resets to the root for the next phrase.

Let *J* denote the number of internal nodes of the parsing tree, including the root. Unique parsability forces M=J(α−1)+1, so

(6) $$J=\frac{M-1}{\alpha -1}.$$

The phrase length satisfies L(y)≥1 for every y∈D, and the codeword length l(y) takes values in $\left\{1,2,\ldots ,l_{\max}\right\}$ for some maximum codeword length $l_{\max}$.

### 2.3. V2v as a Finite-State Encoder

A *finite-state (FS) encoder* over the source alphabet X and output alphabet B is specified by a finite state set Z (of size |Z|), a *next-state function* g:Z×X→Z, and an *output function* $f:Z\times X\to B^{*}$, where $B^{*}$ denotes a set of finite strings over B, possibly including the empty string ε of length zero. For a string $s\in B^{*}$, we write l[s] for its length in bits, with the convention l[ε]=0; this is consistent with l(y) of Section 2 when s=c(y) is the codeword of leaf *y*. When a source sequence $x_{1},x_{2},\ldots$ is fed into the encoder, the state evolves according to the recursion $z_{i+1}=g\left(z_{i},x_{i}\right)$, i=1,2,… (with $z_{1}$ being a fixed initial state), and the encoder outputs the sequence of strings from B, $f\left(z_{1},x_{1}\right),f\left(z_{2},x_{2}\right),\ldots$

The V2V length encoder described in the preceding subsection can be cast as an FS encoder with a state set Z given by the set of *J* internal nodes of the parsing tree (thus |Z|=J), source alphabet X (of cardinality α), and output alphabet B={0,1} (binary codewords). The encoder is in state z∈Z when it has partially matched a phrase and is currently at internal node *z*. For source symbol *x* processed while in state *z*, the next-state and output functions are:

(7) $$g\left(z,x\right)=\left\{\begin{array}{cc} z^{\prime } & \mathrm{if}\;\mathrm{the}\;\mathrm{child}\;\mathrm{of}\;z\;\mathrm{along}\;\mathrm{edge}\;x\;\mathrm{is}\;\mathrm{an}\;\mathrm{internal}\;\mathrm{node}\;z^{\prime },\\ \mathrm{root} & \mathrm{if}\;\mathrm{the}\;\mathrm{child}\;\mathrm{of}\;z\;\mathrm{along}\;\mathrm{edge}\;x\;\mathrm{is}\;a\;\mathrm{leaf}, \end{array}\right.$$

(8) $$f\left(z,x\right)=\left\{\begin{array}{cc} \varepsilon & \;\mathrm{if}\;g(z,x)\neq \mathrm{root},\\ c(y) & \mathrm{if}\;\mathrm{the}\;\mathrm{child}\;\mathrm{of}\;z\;\mathrm{along}\;x\;\mathrm{is}\;\mathrm{leaf}\;y. \end{array}\right.$$

The encoder emits output only when a phrase is completed (i.e., when the child is a leaf), at which point it resets to the root.

**Definition** **1** (Information lossless encoder, [19])**.** *An FS encoder is* information lossless (IL) *if, given the initial state, the output string, and the final state, the input string can be uniquely recovered.*

The IL property holds for uniquely parsable dictionaries, since each codeword maps to a unique leaf, which together with the initial parsing state determines the phrase consumed. For plurally parsable dictionaries [8]—where a source string may have more than one valid parse—the IL property may fail, and the framework below requires modification.

For m≥1, $g(z,x^{m})$ will denote the final state of the encoder when the initial state is *z* and it processes the *m* successive inputs $x^{m}=\left(x_{1},\ldots ,x_{m}\right)\in X^{m}$; $f(z,x^{m})$ denotes the corresponding output string emitted in response to $x^{m}$.

### 2.4. The Generalized Kraft Inequality and a Lower Bound on the Compression Ratio

The classical Kraft–McMillan inequality, $\sum_{y}2^{-l(y)}\leq 1$, characterizes a limitation on lossless codes over a fixed alphabet but does not directly apply to FS encoders. For the IL FS encoder above, the appropriate generalization uses the following matrix.

**Definition** **2** (Kraft matrix, [2])**.** *For an IL FS encoder with state space Z, the Kraft matrix $K\in R^{\left|Z|\times |Z\right|}$ has entries*

(9) $$K_{zz^{\prime }}=\sum_{\left\{x:\;g\left(z,x\right)=z^{\prime }\right\}}2^{-l[f(z,x)]},\;\;\;z,z^{\prime }\in Z,$$

Let spr(K) denote the spectral radius of K. The generalized Kraft inequality reads as follows:

**Theorem** **1** (Generalized Kraft inequality, [2])**.** *For every IL FS encoder, spr(K)≤1.*

**Theorem** **2** (Irreducible bound, [2])**.** *If K is irreducible, then for all $z,z^{\prime }\in Z$ and every integer m≥1,*

(10) $${\left(K^{m}\right)}_{zz^{\prime }}=\sum_{\begin{array}{c} x^{m}:\;g\left(z,x^{m}\right)=z^{\prime } \end{array}}2^{-l[f\left(z,x^{m}\right)]}\leq 2^{\left(J-1\right)l_{\max}}.$$

Note that the right-hand side is independent of *m*. This is the key improvement over the earlier generalized Kraft inequality of Ziv and Lempel [19], whose analogous bound grows linearly in *m*; this linear growth prevents one from extracting any useful rate lower bound from the Ziv–Lempel result.

Substituting J=(M−1)/(α−1) into Theorem 2 and taking the supremum over m≥1 gives the following lower bound on the compression ratio of any V2V length code, valid for any source (not necessarily memoryless); this is Equation (34) of [2], whose proof combines the per-entry bound of Theorem 2 with the standard Kraft-sum-with-slack redundancy bound and is not reproduced here:

**Corollary** **1** (Lower bound on compression ratio)**.** *For an irreducible, uniquely parsable V2V length code with M leaves over an α-ary source alphabet and maximum codeword length $l_{\max}$, the asymptotic compression rate ρ satisfies*

(11) $$\rho \geq \underset{m\geq 1}{\sup}\left[H\left(X_{m}|X^{m-1}\right)-\frac{C(M,\alpha ,l_{\max})}{m}\right],$$

*where $H(X_{m}|X^{m-1})$ is the conditional entropy (in bits per source symbol) of the m-th source symbol given the preceding block $X^{m-1}:=\left(X_{1},\ldots ,X_{m-1}\right)$, and*

(12) $$C\left(M,\alpha ,l_{\max}\right)\overset{\Delta }{=}2\log_{2}J+\left(J-1\right)l_{\max}=2\log_{2}\frac{M-1}{\alpha -1}+\frac{\left(M-\alpha \right)l_{\max}}{\alpha -1}.$$

The right-hand side of Equation (11) cannot be smaller than the entropy rate $H_{\infty }\overset{\Delta }{=}\lim_{m\to \infty }H\left(X_{m}|X^{m-1}\right)$ (a supremum dominates the limit), and decreases in *M*. For a DMS, $H\left(X_{m}|X^{m-1}\right)=H\left(X\right)$, so the supremum is taken over $H\left(X\right)-C\left(M,\alpha ,l_{\max}\right)/m$, which yields H(X). This is a substantially more direct route to the converse than the classical technique of concatenating many phrases and invoking a law-of-large-numbers argument.

### 2.5. Design

Unlike F2V length coding (where Huffman’s algorithm gives a provably optimal greedy construction) and V2F length coding (where Tunstall’s algorithm does the same for memoryless sources), no analogous optimal joint design algorithm is known for V2V length coding, where both the parsing tree and the codeword lengths are simultaneously free. The standard practice is a two-stage heuristic: build a Tunstall-optimal V2F tree, then apply Huffman coding to the leaf probabilities induced by that tree. This heuristic is not known to be jointly optimal and in general it is not [20]. The minimum achievable redundancy for V2V length codes of a given size *M* is an open problem. One known negative result: for a binary memoryless source with 0<P(1)<1/2, no V2V code achieves exactly zero redundancy [3].

The best known achievability result is due to Khodak [3,20]: for any memoryless source there exists a V2V code whose average redundancy decays as $O({\bar{L}}^{-5/3})$, where $\bar{L}$ is the average phrase length. This strictly improves on the $O(1/\bar{L})$ redundancy of the Tunstall–Huffman heuristic, achieved by using a parsing tree that deliberately maintains non-uniform leaf probabilities so that variable codeword lengths can contribute meaningfully. An explicit construction achieving this bound is given in [20].

### 2.6. Notation Summary

The paper accumulates a fair amount of notation as it goes; Table 1 collects the symbols used across more than one section, for reference while reading the more technical parts (Section 3 and Section 5 especially).

## 3. Moments of the Compression Ratio for Memoryless Sources

This section develops the main technical machinery of this paper in two stages. In Section 3.1 and Section 3.2, an exact formula for every integer moment $E\{R_{n}^{k}\}$ is obtained. First, the case k=1 is worked out in full, and then it is generalized for an arbitrary positive integer *k*. Second, from these exact formulas: a refined Edgeworth approximation to the CDF of $R_{n}$ (Section 3.4) that improves on the classical CLT by using the skewness the third moment provides; closed-form O(1/n) asymptotics for the bias and variance via Laplace’s method (Section 3.5); and a large-deviations analysis of $R_{n}$’s tails. A numerical validation against Monte Carlo simulation (Section 3.3) checks the exact formulas along the way. In this section the phrase pairs ${\left(L\left(Y_{i}\right),l\left(Y_{i}\right)\right)}_{i\geq 1}$ are i.i.d. with the same distribution as (L(Y),l(Y)) for a generic phrase *Y*, as is appropriate for a DMS.

### 3.1. The Mean Compression Ratio

We begin with the first moment—the expected compression ratio $E\{R_{n}\}$—as the quantity of primary practical interest and the one that motivates the general approach.

**Theorem** **3.** *For a given DMS and a given V2V length code with a dictionary D and code length function l(·), the expected compression ratio of n phrases is given by*

(13) $$E\left\{R_{n}\right\}=n\cdot \int_{0}^{\infty }\mu_{1}\left(t\right)\cdot {\left[\mu_{0}\left(t\right)\right]}^{n-1}dt.$$

**Proof.**  We use the integral representation 1/s as the Laplace transform of the unit step function, that is, the identity $\frac{1}{s}=\int_{0}^{\infty }e^{-st}dt$, valid for any s>0. Since $\Sigma_{n}=\sum_{i=1}^{n}L\left(Y_{i}\right)$ is a positive integer almost surely, we have

(14) $$R_{n}=\frac{\Lambda_{n}}{\Sigma_{n}}=\Lambda_{n}\cdot \int_{0}^{\infty }e^{-t\Sigma_{n}}dt\;a.s.$$

Taking expectations (justified here by finite-sum linearity, since $\Lambda_{n}$ and $\Sigma_{n}$ take only finitely many values for the finite dictionaries considered throughout this paper, so the outer expectation is itself a finite sum that commutes with the integral term by term), we obtain

(15) $$E\left\{R_{n}\right\}=\int_{0}^{\infty }E\left\{\Lambda_{n}e^{-t\Sigma_{n}}\right\}dt.$$

Now expand

(16) $$\Lambda_{n}e^{-t\Sigma_{n}}=\left[\sum_{i=1}^{n}l\left(Y_{i}\right)\right]\cdot \exp\left[-t\sum_{i=1}^{n}L\left(Y_{i}\right)\right]=\sum_{i=1}^{n}l\left(Y_{i}\right)\prod_{j=1}^{n}e^{-tL(Y_{j})}.$$

Taking the expectation of the *i*-th term and using independence of the phrase pairs,

(17) $$E\left\{l\left(Y_{i}\right)\prod_{j=1}^{n}e^{-tL(Y_{j})}\right\}=E\left\{l\left(Y_{i}\right)e^{-tL(Y_{i})}\right\}\prod_{j\neq i}E\left\{e^{-tL(Y_{j})}\right\}=\mu_{1}\left(t\right){\left[\mu_{0}\left(t\right)\right]}^{n-1}.$$

Since all *n* terms contribute equally (by the i.i.d. assumption), $E\left\{\Lambda_{n}e^{-t\Sigma_{n}}\right\}=n\cdot \mu_{1}\left(t\right){\left[\mu_{0}\left(t\right)\right]}^{n-1}$. Substituting this into Equation (15) and integrating over *t* from 0 to *∞*,

(18) $$E\left\{R_{n}\right\}=n\cdot \int_{0}^{\infty }\mu_{1}\left(t\right)\cdot {\left[\mu_{0}\left(t\right)\right]}^{n-1}dt,$$

which is Equation (13).   □

### 3.2. Extension to General Moments

The proof of Theorem 3 used two ingredients: the Laplace transform representation of 1/s, and independence across phrase pairs. For general k≥1, the same two ingredients apply, but expanding $\Lambda_{n}^{k}$ now produces cross-terms between different phrases, and tracing of these requires some combinatorial bookkeeping. That bookkeeping is organized by *set partitions* of {1,…,k}, defined precisely in the proof below, with a worked example for k=2 and k=3 included along the way.

**Theorem** **4.** *For a given DMS and a given V2V length code with a dictionary D and code length function l(·), the k-th moment of the compression ratio of n phrases is given by*

(19) $$E\left\{R_{n}^{k}\right\}=\frac{1}{(k-1)!}\int_{0}^{\infty }t^{k-1}\sum_{\tau \in P_{k}}\frac{n!}{(n-|\tau |)!}{\left[\mu_{0}\left(t\right)\right]}^{n-|\tau |}\prod_{B\in \tau }\mu_{\left|B\right|}\left(t\right)dt,$$

*where $P_{k}$ denotes the collection of all set partitions of {1,…,k}; |τ| denotes the number of subsets associated with a partition $\tau \in P_{k}$; and |B| denotes the cardinality of a subset B.*

**Proof.**  The integral representation of the function $\frac{1}{s^{k}}$ as a Laplace transform is given by

(20) $$\frac{1}{s^{k}}=\frac{1}{(k-1)!}\int_{0}^{\infty }t^{k-1}e^{-st}dt,\;\;\;\;s>0.$$

Applying this with $s=\Sigma_{n}$ (which is a positive integer a.s.),

(21) $$R_{n}^{k}=\frac{\Lambda_{n}^{k}}{\Sigma_{n}^{k}}=\frac{\Lambda_{n}^{k}}{(k-1)!}\cdot \int_{0}^{\infty }t^{k-1}e^{-t\Sigma_{n}}dt.$$

Taking expectations (again justified by finite-sum linearity, as above), we have

(22) $$E\left\{R_{n}^{k}\right\}=\frac{1}{(k-1)!}\int_{0}^{\infty }t^{k-1}\;E\left\{\Lambda_{n}^{k}e^{-t\Sigma_{n}}\right\}\;dt.$$

It remains to evaluate $E\{\Lambda_{n}^{k}e^{-t\Sigma_{n}}\}$. Now,

(23) $$\Lambda_{n}^{k}={\left[\sum_{i=1}^{n}l\left(Y_{i}\right)\right]}^{k}=\sum_{\left(i_{1},\ldots ,i_{k}\right)\in {\left\{1,\ldots ,n\right\}}^{k}}\prod_{j=1}^{k}l\left(Y_{i_{j}}\right).$$

Each index tuple $\left(i_{1},\ldots ,i_{k}\right)$ induces a set partition τ of the set {1,…,k} to a number of subsets. The slots *a* and *b* in {1,…,k} belong to the same subset pertaining to partition τ if and only if $i_{a}=i_{b}$. We denote by $P_{k}$ for the collection of all set partitions {τ} of {1,…,k} (a finite set; $|P_{1}|=1$, $|P_{2}|=2$, $|P_{3}|=5$), |τ| for the number of blocks of $\tau \in P_{k}$, and |B| for the size of a block B∈τ. (For example, if k=3 and the tuple $\left(i_{1},i_{2},i_{3}\right)=\left(5,7,5\right)$, slots a=1 and b=3 share the phrase index 5 while slot c=2 has the different index 7, so the induced partition is τ={{1,3},{2}}, with |τ|=2 subsets of sizes 2 and 1. The other tuples inducing this same τ are exactly those of the form $\left(j,j^{\prime },j\right)$ for any two *distinct* phrase indices $j\neq j^{\prime }$ in {1,…,n}.) Because the phrase pairs ${\left(L\left(Y_{j}\right),l\left(Y_{j}\right)\right)}_{j\geq 1}$ are i.i.d., the value of $E\{l\left(Y_{i_{1}}\right)\cdots l\left(Y_{i_{k}}\right)e^{-t\Sigma_{n}}\}$ depends on the tuple $\left(i_{1},\ldots ,i_{k}\right)$ only through its induced partition τ, not on the specific phrase indices that realize it. This is what lets us group the $n^{k}$ terms of Equation (23) by partition type, rather than evaluating each term separately. This index-partitioning technique—grouping terms of a power of a sum by which indices coincide, and exploiting exchangeability so that each group’s contribution depends only on the partition it induces—is classical, in the spirit of the partition-lattice approach to moments and cumulants of sums of random variables [21]. What is needed beyond that classical identity is that the exponential weight $e^{-t\Sigma_{n}}$ touches all *n* phrases, not only the *k* appearing in $\Lambda_{n}^{k}$: the n−|τ| phrases outside the partition’s subsets still each contribute a factor (through $\mu_{0}\left(t\right)$, below), and it is this joint accounting—the falling-factorial count together with the untouched phrases’ contribution—that extends the classical moment-of-a-sum computation to the joint quantity $E\{\Lambda_{n}^{k}e^{-t\Sigma_{n}}\}$ actually needed here.

Fix $\tau \in P_{k}$ with blocks $B_{1},\ldots ,B_{\left|\tau \right|}$. A tuple $\left(i_{1},\ldots ,i_{k}\right)$ induces exactly this τ if and only if it assigns the same phrase index to every slot within a given subset, and different phrase indices to slots in different subsets. So realizing τ amounts to choosing an ordered assignment of |τ| distinct phrase indices $j_{1},\ldots ,j_{\left|\tau \right|}\in \left\{1,\ldots ,n\right\}$, one per subset—an injective function from the |τ| blocks to the *n* phrases. Assigning these indices one block at a time, $B_{1}$ may receive any of the *n* phrase indices, $B_{2}$ any of the remaining n−1 (it must differ from $B_{1}$’s), $B_{3}$ any of the remaining n−2, and so on, until $B_{\left|\tau \right|}$, which has n−|τ|+1 choices left; the number of such assignments is the falling factorial $n\left(n-1\right)\cdots \left(n-|\tau |+1\right)=\frac{n!}{(n-|\tau |)!}$.

With blocks $B_{1},\ldots ,B_{\left|\tau \right|}$ assigned distinct phrase indices $j_{1},\ldots ,j_{\left|\tau \right|}$ respectively, the product $l\left(Y_{i_{1}}\right)\cdots l\left(Y_{i_{k}}\right)$ collapses to $\prod_{r=1}^{\left|\tau \right|}{\left[l\left(Y_{j_{r}}\right)\right]}^{|B_{r}|}$, since subset $B_{r}$ contributes $|B_{r}|$ copies of the same factor $l(Y_{j_{r}})$. Splitting $e^{-t\Sigma_{n}}=\prod_{j=1}^{n}e^{-tL(Y_{j})}$ into the |τ| marked phrases $j_{1},\ldots ,j_{\left|\tau \right|}$ and the remaining n−|τ| phrases, and using independence across phrases,

(24) $$\begin{array}{cc} E\left\{\prod_{a=1}^{k}l\left(Y_{i_{a}}\right)\cdot \prod_{j=1}^{n}e^{-tL(Y_{j})}\right\} & =\prod_{r=1}^{\left|\tau \right|}E\left\{{\left[l\left(Y_{j_{r}}\right)\right]}^{|B_{r}|}e^{-tL(Y_{j_{r}})}\right\}\cdot \prod_{j\notin \{j_{1},\ldots ,j_{\left|\tau \right|}\}}E\left\{e^{-tL(Y_{j})}\right\}\\ \; & =\prod_{B\in \tau }\mu_{\left|B\right|}\left(t\right)\cdot {\left[\mu_{0}\left(t\right)\right]}^{n-|\tau |}, \end{array}$$

using the i.i.d. property and the definitions of $\mu_{j}\left(t\right)$ and $\mu_{0}\left(t\right)$. This value depends only on τ (through its block sizes), not on the specific phrases $j_{1},\ldots ,j_{\left|\tau \right|}$ chosen to realize it—consistent with the partition-dependence noted above.

Every $\tau \in P_{k}$ contributes the same per-tuple expectation, as just shown, so summing over all $\frac{n!}{(n-|\tau |)!}$ tuples that induce it (the falling-factorial count derived above) and then over all $\tau \in P_{k}$ gives

(25) $$E\left\{\Lambda_{n}^{k}e^{-t\Sigma_{n}}\right\}=\sum_{\tau \in P_{k}}\frac{n!}{(n-|\tau |)!}{\left[\mu_{0}\left(t\right)\right]}^{n-|\tau |}\prod_{B\in \tau }\mu_{\left|B\right|}\left(t\right).$$

Substituting into Equation (22) gives Equation (19).  □

For k=2 and k=3, the integrals are explicitly

(26) $$E\left\{R_{n}^{2}\right\}=\int_{0}^{\infty }t\left(n\mu_{2}\left(t\right){\left[\mu_{0}\left(t\right)\right]}^{n-1}+n\left(n-1\right){\left[\mu_{1}\left(t\right)\right]}^{2}{\left[\mu_{0}\left(t\right)\right]}^{n-2}\right)dt,$$

(27) $$\begin{array}{cc} E\left\{R_{n}^{3}\right\}=\frac{1}{2}\int_{0}^{\infty }t^{2} & (n\mu_{3}\left(t\right){\left[\mu_{0}\left(t\right)\right]}^{n-1}+3n\left(n-1\right)\mu_{2}\left(t\right)\mu_{1}\left(t\right){\left[\mu_{0}\left(t\right)\right]}^{n-2} \end{array}$$

(28) $$\begin{array}{cc} \; & +n\left(n-1\right)\left(n-2\right){\left[\mu_{1}\left(t\right)\right]}^{3}{\left[\mu_{0}\left(t\right)\right]}^{n-3})dt. \end{array}$$

From these three moments one obtains the mean, variance, and skewness of $R_{n}$ via $\mathrm{Var}\left\{R_{n}\right\}=E\left\{R_{n}^{2}\right\}-{\left(E\left\{R_{n}\right\}\right)}^{2}$ and $\gamma_{1}=\left(E\left\{R_{n}^{3}\right\}-3E\left\{R_{n}\right\}E\left\{R_{n}^{2}\right\}+2{\left(E\left\{R_{n}\right\}\right)}^{3}\right)/{\left(\mathrm{Var}\left\{R_{n}\right\}\right)}^{3/2}$.

### 3.3. Validation

We now validate Equation (19) on a simple V2V length code: a binary memoryless source with P(0)=1−P(1)=p=0.8, parsed by the Tunstall tree with dictionary {00,01,1}, and coded with the Huffman assignment (l(00),l(01),l(1))=(1,2,2). The same source, tree, and codeword assignment are used again for the bias-decomposition example in Section 4. This is a complete prefix code, $2^{-1}+2^{-2}+2^{-2}=1$, so Kraft’s inequality holds with equality, and every codeword length is a positive integer. The phrase probabilities are $Q\left(00\right)=p^{2}=0.64$, Q(01)=p(1−p)=0.16, Q(1)=1−p=0.2, giving $\bar{L}=1.8$, $\bar{l}=1.36$, and $\rho =\bar{l}/\bar{L}=0.7556$.

For this model, $\mu_{j}\left(t\right)=E\left\{{\left[l\left(Y\right)\right]}^{j}e^{-tL(Y)}\right\}$ is the explicit three-term sum

(29) $$\mu_{j}\left(t\right)=0.64\cdot 1^{j}e^{-2t}+0.16\cdot 2^{j}e^{-2t}+0.2\cdot 2^{j}e^{-t},$$

so, e.g., $\mu_{0}\left(t\right)=0.8e^{-2t}+0.2e^{-t}$ and $\mu_{1}\left(t\right)=0.96e^{-2t}+0.4e^{-t}$. The three integrals, Equations (13), (26) and (27), are evaluated by standard numerical quadrature (Gaussian quadrature after the substitution t=u/n to handle the concentration near t=0 for larger *n*).

At n=50 phrases, the exact formulas give

(30) $$E\left\{R_{50}\right\}=0.7571,\;\mathrm{Var}\left\{R_{50}\right\}=0.003230,\;\gamma_{1}=0.2878.$$

For validation, we performed a Monte Carlo simulation of 1,000,000 independent realizations of 50 phrase pairs $\left(L\left(Y_{i}\right),l\left(Y_{i}\right)\right)$, drawn according to the three phrase probabilities above, computing the realized ratio $R_{50}=\Lambda_{50}/\Sigma_{50}$ for each. The empirical estimates are

(31) $$\hat{E\{R_{50}\}}=0.7571,\;\hat{\mathrm{Var}\{R_{50}\}}=0.003226,\;\hat{\gamma_{1}}=0.2875.$$

Agreement is to three or four significant figures throughout—the skewness $\gamma_{1}$ in particular is the first quantity that Equation (27) provides beyond what a first-order approximation would give—and the exact formulas require no simulation effort at all.

### 3.4. The Edgeworth Approximation

The three exact moment formulas, Equations (13)–(27), can be used to construct a sharper approximation to the CDF of $R_{n}$ than the ordinary CLT (Gaussian) approximation. By the bivariate CLT, the pair $\left(\Lambda_{n}-n\bar{l},\Sigma_{n}-n\bar{L}\right)/\sqrt{n}$ converges in distribution to a bivariate normal as n→∞. By the delta method [1] (Theorem 3.1), any function that is continuously differentiable at the limiting mean again yields a normal limit, with asymptotic variance determined by the gradient of the function and the full limiting covariance matrix. Applied to the map (λ,m)↦λ/m, which is continuously differentiable at $\left(\bar{l},\bar{L}\right)$ since $\bar{L}>0$, this implies that $Z_{n}\overset{\Delta }{=}\left(R_{n}-E\left\{R_{n}\right\}\right)/\sqrt{\mathrm{Var}\{R_{n}\}}$ converges in distribution to a standard normal N(0,1). The CLT approximation to the CDF $F\left(x\right)\overset{\Delta }{=}\mathrm{Pr}\left\{R_{n}\leq x\right\}$ is therefore $\Phi_{\mathrm{nor}}\left(z\right)$, where $z=\left(x-E\left\{R_{n}\right\}\right)/\sqrt{\mathrm{Var}\{R_{n}\}}$ and $\Phi_{\mathrm{nor}}$ is the standard normal CDF. But this approximation ignores the skewness of the distribution, which is $O(1/\sqrt{n})$ and non-negligible at moderate *n*.

The one-term Edgeworth expansion corrects for the skewness. It approximates *F* by

(32) $$F_{\mathrm{Edgeworth}}\left(x\right)\overset{\Delta }{=}\Phi_{\mathrm{nor}}\left(z\right)-\varphi \left(z\right)\;\frac{\gamma_{1}}{6}\left(z^{2}-1\right),$$

where $z=\left(x-E\left\{R_{n}\right\}\right)/\sqrt{\mathrm{Var}\{R_{n}\}}$, $\varphi =\Phi_{\mathrm{nor}}^{\prime }$ is the standard normal density, and $\gamma_{1}$ is the skewness of $R_{n}$. The approximation, Equation (32), is a standard result in the theory of Edgeworth expansions for sums of i.i.d. random variables [22] (Ch. XVI); its validity for the ratio $R_{n}=\Lambda_{n}/\Sigma_{n}$ follows from the delta method applied to the bivariate CLT for $\left(\Lambda_{n},\Sigma_{n}\right)$, under standard moment conditions on the phrase pair (L(Y),l(Y)). The key point is that all three parameters entering Equation (32)—$E\{R_{n}\}$, $\mathrm{Var}\{R_{n}\}$, and $\gamma_{1}$—are available from Equations (13)–(27) for every finite *n*.

Continuing the same numerical example as in Section 3.3, Table 2 shows the absolute approximation error $|F\left(x\right)-F_{\mathrm{Edgeworth}}\left(x\right)|$ for n=50, at several standardized values *z*, comparing the CLT approximation against Equation (32) with the exact $\gamma_{1}=0.2878$.

The Edgeworth correction reduces the approximation error at every tabulated point, by a factor ranging from about 3 to nearly 200 depending on *z*; averaged over the range shown, the mean absolute error drops by a factor of about 3.4 and the maximum error by a factor of about 2.7. This is a more modest improvement than a smooth, continuously-supported distribution would show (compare Remark 1 below), since L(Y) and l(Y) each take only two values here, but it is a genuine improvement for an actual code.

**Figure 1:**
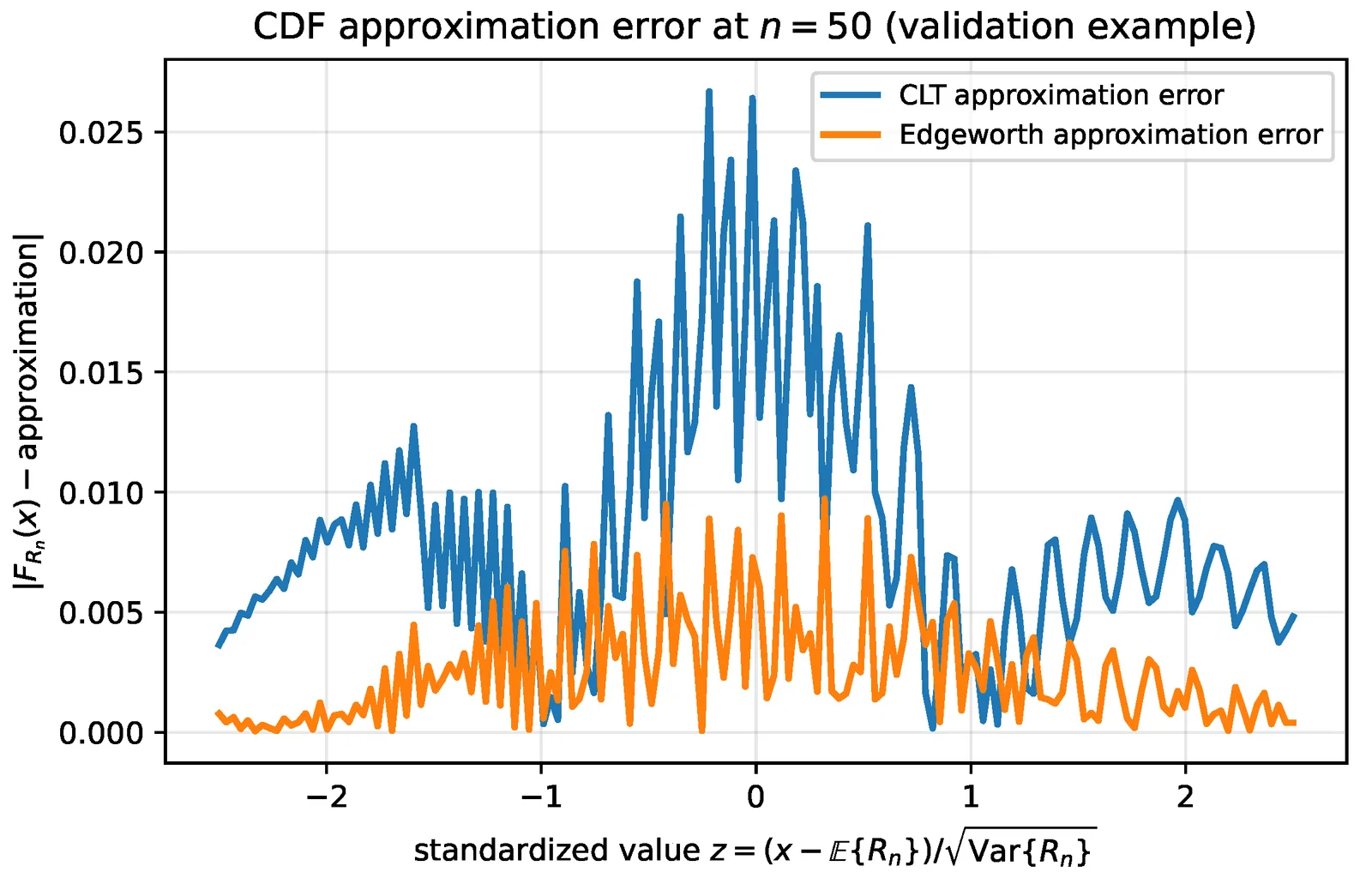
Absolute CDF approximation error, CLT vs. Edgeworth equation, Equation (32), as a function of the standardized value *z*, for the validation example of Section 3.3 at n=50. Ground truth is a 20,000,000-trial Monte Carlo simulation.

Figure 1 shows this comparison as a continuous function of *z* rather than at the five tabulated points, against a 20,000,000-trial Monte Carlo baseline. The CLT error traces out a broad envelope peaking near z=0, while the Edgeworth error stays consistently lower across the entire range shown. Both curves show some oscillation rather than a perfectly smooth profile; this is a genuine finite-alphabet effect discussed in Remark 1 below.

**Remark** 1
(A finite-alphabet effect)**.** *Because L(Y)∈{1,2} and l(Y)∈{1,2} each take only two values, $\Lambda_{n}$ and $\Sigma_{n}$ are both sums of small integers, so $R_{n}=\Lambda_{n}/\Sigma_{n}$ takes values in a finite, coarsely-spaced set of rationals for any fixed n—its exact CDF is a step function with comparatively few, comparatively large jumps. Neither the CLT nor the Edgeworth approximation is built to track a step function’s exact jumps (both are continuous-x approximations, designed for the lattice spacing to vanish as n→∞), so both error curves in Figure 1 inherit some oscillation from this discreteness, on top of their genuine approximation error. This is a real feature of finite, small-dictionary V2V codes, not an artifact: a synthetic phrase-length law with a smooth, unbounded, or finely-spaced support—as used in an earlier version of this example—would mask it entirely. The Edgeworth correction still reduces the error at every point tested, as Table 2 shows; it is simply a less clean improvement than the idealized, non-lattice case.*

### 3.5. Asymptotic Approximation via the Laplace Method of Integration

For large *n*, the integrands in Equation (19) are sharply concentrated near t=0 and can be evaluated asymptotically by the Laplace method of integration. The change of integration variabe t=u/n transforms the integrals into a form where the concentration is explicit and a systematic expansion in 1/n can be carried out. There are two versions of Laplace’s method for an integral $\int_{a}^{b}g\left(t\right)e^{-nf(t)}dt$, depending on where *f* attains its minimum over the domain of integration. If the minimum occurs at an interior point $t_{0}\in \left(a,b\right)$ with $f^{\prime }\left(t_{0}\right)=0$, the local behavior of *f* near $t_{0}$ is quadratic, and so, $e^{-nf(t)}$ is locally approximated by a Gaussian, $e^{-nf(t_{0})}\cdot \exp\left\{-\frac{nf^{\prime \prime }\left(t_{0}\right)}{2}{\left(t-t_{0}\right)}^{2}\right\}$, giving the familiar $\sqrt{\frac{2\pi }{nf^{\prime \prime }\left(t_{0}\right)}}$ prefactor. If instead the minimum occurs at a boundary point of the domain (*a* or *b*) with $f^{\prime }\left(t_{0}\right)\neq 0$, the local behavior of *f* is linear rather than quadratic, and $e^{-nf(t)}$ is locally approximated at the vicinity of $t_{0}$ by a plain exponential, $e^{-nf(t_{0})}e^{-nf^{\prime }\left(t_{0}\right)\left(t-t_{0}\right)}$, rather than a Gaussian; its moments against a power series in $\left(t-t_{0}\right)$ are then elementary Gamma-function integrals rather than Gaussian moments (see, e.g., ref. [23] (Section 4.3) or ref. [24] (p. 48)). This second, boundary case is the one relevant here: as shown below, $f\left(t\right)\overset{\Delta }{=}-\ln\mu_{0}\left(t\right)$ attains its minimum over t∈[0,∞) at the boundary point $t_{0}=0$ with $f^{\prime }\left(0\right)=\bar{L}\neq 0$, so ${\left[\mu_{0}\left(t\right)\right]}^{n}=e^{-nf(t)}$ is approximated near t=0 by the exponential $e^{-n\bar{L}t}$. The derivation below carries this out to one further order in 1/n than the leading term alone provides, since the bias coefficient *C* requires the O(1/n) correction.

Let $f\left(t\right)=-\ln\mu_{0}\left(t\right)$ denote the negative cumulant generating function (CGF) of −L(Y). Since L(Y)≥1 a.s., f(0)=0 and $f^{\prime }\left(0\right)=E\left\{L\left(Y\right)\right\}=\bar{L}>0$. Furthermore, *f* is strictly concave as $f^{\prime \prime }\left(t\right)<0$ for all t≥0. In particular, f(t)>0 for all t>0, so ${\left[\mu_{0}\left(t\right)\right]}^{n-1}=e^{-(n-1)f(t)}\to 0$ exponentially fast in *n* for every fixed t>0. The integrand of Equation (13), which is proportional to $\mu_{1}\left(t\right){\left[\mu_{0}\left(t\right)\right]}^{n-1}$, therefore concentrates near $t_{0}=0$ as n→∞. Substituting t=u/n gives ${\left[\mu_{0}\left(u/n\right)\right]}^{n-1}=e^{-(n-1)f(u/n)}\approx e^{-\bar{L}u}$ (to leading order for large *n*), and after the substitution the integral becomes $\int_{0}^{\infty }\mu_{1}\left(u/n\right)e^{-(n-1)f(u/n)}du$, which is O(1).

*The bias term.* Write $\bar{L}=E\left\{L\left(Y\right)\right\}$, $\kappa_{2}=\mathrm{Var}\left\{L\left(Y\right)\right\}$, $\bar{l}=E\left\{l\left(Y\right)\right\}$, $c_{lL}=E\left\{l\left(Y\right)L\left(Y\right)\right\}$. The O(1/n) expansion of $E\{R_{n}\}$ follows from a general fact about this boundary case of the Laplace method, stated here and proved in Appendix A (see also ref. [23] (Section 4.3) and ref. [24] (p. 48) for the general theory).

**Lemma** 1
(Boundary Laplace expansion)**.** *Let f:[0,∞)→[0,∞) satisfy f(0)=0, be twice continuously differentiable at t=0 with $f^{\prime }\left(0\right)=a$ for some a>0 and $f^{\prime \prime }\left(0\right)=-b$, and let h be continuously differentiable at t=0 with $h\left(0\right)=h_{0}$, $h^{\prime }\left(0\right)=h_{1}$ (both suitably regular for the expansion below to hold, as verified for $f=-\ln\mu_{0}$ above). Then, for any integers k≥1 and m≥1, as n→∞,*

$$\begin{array}{cc} & \frac{n!}{(n-m)!}\int_{0}^{\infty }t^{k-1}h\left(t\right)e^{-(n-m)f(t)}dt\\ = & n^{m-k}\left[\frac{\left(k-1\right)!h_{0}}{a^{k}}+\frac{(k-1)!}{2na^{k+2}}\left(a^{2}h_{0}m\left(2k-m+1\right)+2ah_{1}k+bh_{0}k\left(k+1\right)\right)+O\left(\frac{1}{n^{2}}\right)\right]. \end{array}$$

The case k=m=1, h=g reduces to

$$n\cdot \int_{0}^{\infty }g\left(t\right)e^{-(n-1)f(t)}dt=\frac{g_{0}}{a}+\frac{a^{2}g_{0}+ag_{1}+bg_{0}}{na^{3}}+O\left(\frac{1}{n^{2}}\right),$$

the form used just below.

Applying Lemma 1 with $f\left(t\right)=-\ln\mu_{0}\left(t\right)$ and $g=\mu_{1}$, from the previous paragraph, $a=\bar{L}$ and $b=\kappa_{2}$; and since $\mu_{1}\left(0\right)=E\left\{l\left(Y\right)\right\}=\bar{l}$ and

(33) $$\mu_{1}^{\prime }\left(t\right)=\frac{d}{dt}E\left\{l\left(Y\right)e^{-tL(Y)}\right\}=-E\left\{L\left(Y\right)l\left(Y\right)e^{-tL(Y)}\right\},$$

so $\mu_{1}^{\prime }\left(0\right)=-c_{lL}$, we have $g_{0}=\bar{l}$ and $g_{1}=-c_{lL}$. Substituting into Equation (33),

(34) $$E\left\{R_{n}\right\}=n\cdot \int_{0}^{\infty }\mu_{1}\left(t\right){\left[\mu_{0}\left(t\right)\right]}^{n-1}dt=\frac{\bar{l}}{\bar{L}}+\frac{1}{n}\cdot \frac{{\bar{L}}^{2}\bar{l}-\bar{L}c_{lL}+\kappa_{2}\bar{l}}{{\bar{L}}^{3}}+O\left(\frac{1}{n^{2}}\right)=\rho +\frac{C}{n}+O\left(\frac{1}{n^{2}}\right)$$

with

(35) $$C=\frac{\bar{l}\left({\bar{L}}^{2}+\kappa_{2}\right)-c_{lL}\bar{L}}{{\bar{L}}^{3}}.$$

We state this as Proposition 1 below.

**Proposition** **1** (Asymptotic bias)**.**

(36) $$\lim_{n\to \infty }n\cdot \left(E\left\{R_{n}\right\}-\rho \right)=\frac{\bar{l}\left({\bar{L}}^{2}+\kappa_{2}\right)-c_{lL}\bar{L}}{{\bar{L}}^{3}}\overset{\Delta }{=}C,$$

*where $\rho =\bar{l}/\bar{L}$.*

A comment is in order concerning an alternative route for calculating the bias—the delta-method. Since ${\bar{U}}_{n}:=\Sigma_{n}/n$ and ${\bar{V}}_{n}:=\Lambda_{n}/n$ are exact sample means of i.i.d. data, $E\left\{{\bar{U}}_{n}\right\}=\bar{L}$ and $E\left\{{\bar{V}}_{n}\right\}=\bar{l}$ exactly, for every *n*. Writing g(u,v):=v/u and Taylor-expanding $R_{n}=g\left({\bar{U}}_{n},{\bar{V}}_{n}\right)$ to second order around $\left(\bar{L},\bar{l}\right)$, the first-derivative terms vanish upon taking expectations, since the sample means are exactly unbiased—so the entire bias comes from the second derivatives:

(37) $$C=\frac{1}{2}\left(g_{uu}\mathrm{Var}\left\{L\right\}+2g_{uv}\mathrm{Cov}\left\{L,l\right\}\right)=\frac{\rho \;\mathrm{Var}\{L\}-\mathrm{Cov}\{L,l\}}{{\bar{L}}^{2}},$$

using $g_{uu}=2\rho /{\bar{L}}^{2}$, $g_{uv}=-1/{\bar{L}}^{2}$, $g_{vv}=0$ at $\left(\bar{L},\bar{l}\right)$—algebraically identical to Equation (36), with $\mathrm{Var}\left\{L\right\}=\kappa_{2}$ and $\mathrm{Cov}\left\{L,l\right\}=c_{lL}-\bar{L}\bar{l}$.

This is a useful independent cross-check, but not a substitute for the derivation above. The delta method, by construction, gives only the leading O(1/n) correction: it starts from an asymptotic expansion of $R_{n}$ itself and stops at second order once that correction is in hand, since the first-derivative terms vanish identically and nothing forces the expansion any further. The Laplace-method route above instead starts from the exact formula Equation (13), valid at every finite *n*, and reaches the bias by expanding that formula asymptotically—so the same machinery, carried one order further, would give the $O(1/n^{2})$ term too, and (via Theorem 4 and Lemma 1 applied repeatedly, as just below) gives every higher moment—variance, skewness, and beyond—from a single combinatorial formula, rather than requiring a fresh, increasingly delicate multivariate Taylor expansion for each one in turn. It also extends uniformly to the Markov case of Section 5: there, the delta method’s natural generalization requires tracking how a phrase’s own fluctuation correlates with the state it leaves the chain in—a genuine complication with no analogue here—whereas an eigenvalue-perturbation argument handles this automatically, by perturbing the joint spectral object directly rather than decomposing the bias into pieces by hand.

*The variance term.* Lemma 1, applied twice more, yields the asymptotic variance directly. Write $E\left\{R_{n}^{2}\right\}=I_{1}+I_{2}$ where
  (38) $$I_{1}=\int_{0}^{\infty }tn\mu_{2}\left(t\right){\left[\mu_{0}\left(t\right)\right]}^{n-1}dt,\;I_{2}=\int_{0}^{\infty }tn\left(n-1\right){\left[\mu_{1}\left(t\right)\right]}^{2}{\left[\mu_{0}\left(t\right)\right]}^{n-2}dt,$$
  each matching the Lemma’s left-hand side with k=2 (since the extra factor is $t=t^{k-1}$). For $I_{1}$, m=1 and $h=\mu_{2}$, so $h_{0}=\mu_{2}\left(0\right)=s_{l2}$ where $s_{l2}:=E\left\{{\left[l\left(Y\right)\right]}^{2}\right\}$; since $I_{1}$ itself turns out to contribute only at order 1/n to $E\{R_{n}^{2}\}$, only the Lemma’s leading term is needed:
  (39) $$I_{1}=\frac{s_{l2}}{n{\bar{L}}^{2}}+O\left(\frac{1}{n^{2}}\right).$$

For $I_{2}$, m=2 and $h={\left[\mu_{1}\right]}^{2}$, so $h_{0}={\left[\mu_{1}\left(0\right)\right]}^{2}={\bar{l}}^{2}$ and, by the product rule, $h_{1}=2\mu_{1}\left(0\right)\mu_{1}^{\prime }\left(0\right)=-2\bar{l}\;c_{lL}$; since $I_{2}$ contributes at leading order O(1), both terms of the Lemma are needed:

(40) $$I_{2}=\frac{{\bar{l}}^{2}}{{\bar{L}}^{2}}+\frac{1}{n}\cdot \frac{\bar{l}\left(3{\bar{L}}^{2}\bar{l}-4\bar{L}\;c_{lL}+3\kappa_{2}\bar{l}\right)}{{\bar{L}}^{4}}+O\left(\frac{1}{n^{2}}\right).$$

**Proposition** **2** (Asymptotic variance)**.** *With $s_{l2}=E\left\{{\left[l\left(Y\right)\right]}^{2}\right\}$:*

(41) $$\lim_{n\to \infty }n\cdot \mathrm{Var}\left\{R_{n}\right\}=\frac{{\bar{L}}^{2}\left(s_{l2}-{\bar{l}}^{2}\right)-2\bar{L}\bar{l}\left(c_{lL}-\bar{L}\bar{l}\right)+\kappa_{2}{\bar{l}}^{2}}{{\bar{L}}^{4}}.$$

**Proof.**  Adding $I_{1}$ and $I_{2}$ above gives $E\{R_{n}^{2}\}$ to O(1/n); subtracting ${\left(E\left\{R_{n}\right\}\right)}^{2}=\rho^{2}+2\rho C/n+O\left(n^{-2}\right)$ (squaring Proposition 1) and multiplying by *n*, the O(1) terms cancel exactly (since ${\bar{l}}^{2}/{\bar{L}}^{2}=\rho^{2}$), leaving Equation (41) after collecting the remaining terms over the common denominator ${\bar{L}}^{4}$.  □

On the validation example of Section 3.3, the analytic formulas of Propositions 1 and 2 evaluate to a bias coefficient of 0.076818 and an asymptotic scaled variance of 0.159000. The exact formula Equation (19) evaluated at large *n* gives $n(E\left\{R_{n}\right\}-\rho )\to 0.076818$ and $n\mathrm{Var}\{R_{n}\}\to 0.159000$, matching to six significant figures and confirming both propositions numerically.

**Remark** **2** (Design implication)**.** *Proposition 1 shows that for a fixed parsing tree (hence fixed $\bar{L}$ and Var{L}), the bias $E\{R_{n}\}-\rho$ is minimized (i.e., $E\{R_{n}\}$ converges to ρ fastest) when Cov{L,l} is maximized. This has a clear design interpretation: longer phrases should be assigned longer codewords; i.e., Cov{L,l} should be made positive. Whether a specific codeword assignment achieves this depends on the source and tree; the bias formula gives a precise, finite-n quantification of how far any given code deviates from the optimum. Section 4 makes this concrete: the Khodak construction’s near-Shannon codeword assignment achieves Cov{L,l}≈HVar{L}>0, a direct consequence of matching codeword length to phrase length via the source entropy, and this is exactly the mechanism by which it improves on Tunstall–Huffman—whose codeword assignment instead minimizes ρ directly, with no regard for this covariance, and can in fact drive it negative, as shown numerically there.*

### 3.6. Large Deviations of the Compression Ratio

The Edgeworth expansion of Section 3.4 characterizes the behavior of $R_{n}$ in the $O(1/\sqrt{n})$ fluctuation region around ρ. For deviations of fixed size Δ>0 (independent of *n*), the probability $\mathrm{Pr}\{R_{n}>\rho +\Delta \}$ decays exponentially in *n*, and its exact exponential rate follows directly from Cramér’s theorem.

The key observation is that the event $\left\{R_{n}>\rho +\Delta \right\}$ is equivalent to a standard large-deviations event for a sum of i.i.d. random variables. Since

(42) $$R_{n}>\rho +\Delta \;\Leftrightarrow \;\frac{\Lambda_{n}}{\Sigma_{n}}>\rho +\Delta \;\Leftrightarrow \;\sum_{i=1}^{n}\left[l\left(Y_{i}\right)-\left(\rho +\Delta \right)L\left(Y_{i}\right)\right]>0,$$

the event is determined by whether the partial sum of the i.i.d. random variables $Z_{i}^{\left(\Delta \right)}:=l\left(Y_{i}\right)-\left(\rho +\Delta \right)L\left(Y_{i}\right)$ exceeds zero. Since $E\left\{Z_{i}^{\left(\Delta \right)}\right\}=E\left\{l\left(Y\right)\right\}-\left(\rho +\Delta \right)E\left\{L\left(Y\right)\right\}=-\Delta E\left\{L\left(Y\right)\right\}<0$ for Δ>0, this is a large-deviations event with the sum crossing zero against its drift. An identical reduction handles the lower tail: $\left\{R_{n}<\rho -\Delta \right\}\Leftrightarrow \sum_{i}\left[l\left(Y_{i}\right)-\left(\rho -\Delta \right)L\left(Y_{i}\right)\right]<0$, with E{l(Y)−(ρ−Δ)L(Y)}=ΔE{L(Y)}>0.

By Cramér’s theorem, for every Δ>0 (all logarithms in this subsection are natural logarithms),

(43) $$\begin{array}{cc} \frac{1}{n}\log\mathrm{Pr}\left\{R_{n}>\rho +\Delta \right\} & \;\to \;-I_{+}\left(\Delta \right), \end{array}$$

(44) $$\begin{array}{cc} \frac{1}{n}\log\mathrm{Pr}\left\{R_{n}<\rho -\Delta \right\} & \;\to \;-I_{-}\left(\Delta \right), \end{array}$$

where the rate functions are

(45) $$I_{+}\left(\Delta \right)\;=\;\underset{\theta >0}{\sup}\left\{-\log E\left\{e^{\theta [l-(\rho +\Delta )L]}\right\}\right\},$$

(46) $$I_{-}\left(\Delta \right)\;=\;\underset{\theta <0}{\sup}\left\{-\log E\left\{e^{\theta [l-(\rho -\Delta )L]}\right\}\right\}.$$

For a real parameter θ and constant *c*, let $P_{\theta }^{\left(c\right)}$ denote the exponentially tilted law of (L,l) obtained by weighting each realization proportionally to $e^{\theta (l-cL)}$, and write $E_{\theta }^{\left(c\right)}\left\{\cdot \right\}$ for the corresponding expectation; below, *c* is ρ+Δ for the upper tail and ρ−Δ for the lower tail, and we abbreviate $P_{\theta }^{\left(c\right)}$ and $E_{\theta }^{\left(c\right)}$ by $P_{\theta }$ and $E_{\theta }$ since *c* is clear from context. The supremum over θ>0 in $I_{+}$ is unconstrained (the CGF is finite for all θ>0 provided *ℓ* has all exponential moments, which holds for a finite alphabet); for a finite dictionary *L* is bounded, so the CGF of l−(ρ−Δ)L is finite for all θ∈R, and the supremum over θ<0 in $I_{-}$ is unconstrained; for codes with unbounded phrase lengths, the supremum may be constrained to an interval $\left(\theta_{\min},0\right)$. The supremum in each case is achieved at a unique $\theta^{*}$. For the upper tail, $\theta^{*}>0$ is the unique positive root of

(47) $$\frac{E\{\left[l-\left(\rho +\Delta \right)L\right]\;e^{\theta^{*}\left[l-\left(\rho +\Delta \right)L\right]}\}}{E\{e^{\theta^{*}\left[l-\left(\rho +\Delta \right)L\right]}\}}=0,$$

and for the lower tail, $\theta^{*}<0$ is the unique negative root of

(48) $$\frac{E\{\left[l-\left(\rho -\Delta \right)L\right]\;e^{\theta^{*}\left[l-\left(\rho -\Delta \right)L\right]}\}}{E\{e^{\theta^{*}\left[l-\left(\rho -\Delta \right)L\right]}\}}=0.$$

In both cases the condition expresses that the compression ratio under the tilted distribution $P_{\theta^{*}}$ equals the target level: $E_{\theta^{*}}\left\{l\right\}/E_{\theta^{*}}\left\{L\right\}=\rho +\Delta$ (upper tail) or ρ−Δ (lower tail). Thus $P_{\theta^{*}}$ is the unique exponential tilt of the original phrase-pair law under which the large-deviations event is typical.

The rate functions $I_{+},I_{-}$ are not built from a separate toolkit: $E\left\{e^{\theta [l-(\rho +\Delta )L]}\right\}=\mu \left(\theta ,t\right)$ along the ray t=θ(ρ+Δ) (and similarly for $I_{-}$ along t=θ(ρ−Δ)), the same joint moment generating function introduced in Section 1.1, which reduces to $\mu_{0}\left(t\right)$ at θ=0 and to $\mu_{1}\left(t\right)$ upon differentiating in θ at θ=0 (Section 2). The exact moments of Section 3 and the large-deviations rate functions here are two different slices of this same underlying two-parameter family, evaluated in different regimes: *t* alone for the moments, and along the rays above for the tails.

## 4. Comparison with V2F and F2V Length Codes

The three code families differ in which of the phrase length *L* and codeword length *ℓ* they allow to vary: F2V fixes *L*, V2F fixes *ℓ*, and V2V lets both vary. The fact that V2V can match or beat the other two on the asymptotic rate ρ is not surprising—it strictly contains them as special cases. The more informative question is what this extra freedom does to the finite-*n* behavior captured by *C*, and what, if anything, it reveals about why some V2V constructions substantially outperform both alternatives while others do not.

*Setup.* Fix a binary memoryless source with p=P(0)=0.8 (H=0.722 bits/symbol) and the Tunstall tree {00,01,1} ($\bar{L}=1.8$, Var{L}=0.16).
*The structural decomposition.* The bias formula of Proposition 1 rewrites as
  (49) $$C=\frac{\rho \;\mathrm{Var}\{L\}-\mathrm{Cov}\{L,l\}}{{\bar{L}}^{2}},$$
  which separates cleanly by code family:
  - F2V: *L* is deterministic, so Var{L}=Cov{L,l}=0 and C=0 identically—not a design achievement, but a triviality of having no phrase-length randomness to create bias from. It buys nothing for ρ.
  - V2F: *ℓ* is constant, so Cov{L,l}=0 always, giving $C=\rho \mathrm{Var}\left\{L\right\}/{\bar{L}}^{2}>0$ with no freedom to reduce it: the fixed codeword length that limits ρ to $O(1/\bar{L})$ redundancy is the same constraint that locks in this bias.
  - V2V: Both vary, so Cov{L,l} is a genuine design variable. Correlating *L* and *ℓ* positively—longer phrases getting longer codewords—reduces *C* below what V2F could achieve with the same tree and the same Var{L}.

This freedom cuts both ways, though: the Huffman codeword assignment that minimizes ρ need not create positive covariance. For the tree above, Huffman assigns the shortest codeword to the most probable phrase (00, P=0.64), which happens to be one of the longest phrases (L=2), giving Cov{L,l}=−0.128<0 and $C_{V2V}=0.077$—larger than $C_{V2F}=0.055$ despite $\rho_{V2V}=0.756<\rho_{V2F}=1.111$. The same tree admits a different codeword assignment (giving the shortest codeword to the shortest phrase instead) that achieves Cov{L,l}=+0.160 and C=0 exactly, at the cost of a worse ρ=1. V2F and F2V have no such choice to make; V2V’s freedom is precisely the ability to trade between these two objectives, in either direction.

*Why this matters: the Khodak code.* The tension above raises the question of whether ρ and *C* can be improved together rather than traded off, and the Khodak code [3,20] shows that they can. By the conservation of entropy [25], $H\left(Y\right)=\bar{L}\;H$ for any parsing tree, where H(Y) is the phrase entropy; the Khodak construction assigns near-Shannon codewords, $l\left(y\right)\approx -\log_{2}Q\left(y\right)$, to a tree deliberately built to preserve non-uniform leaf probabilities. By the asymptotic equipartition property, a typical phrase of length L(y) satisfies $Q\left(y\right)\approx 2^{-L(y)H}$, so l(y)≈L(y)H holds for the typical phrases that carry essentially all the probability mass as L(y) grows—not for every individual leaf (a phrase consisting entirely of the single most probable symbol, for instance, gets a codeword far shorter than L(y)H), but for enough of the distribution to drive the averages below. This achieves redundancy $r:=\rho -H=O({\bar{L}}^{-5/3})$—strictly better than the $O(1/\bar{L})$ available to V2F or to Tunstall–Huffman. The same typical-phrase approximation gives Cov{L,l}≈HVar{L}, so substituting into Equation (49),
  (50) $$C\;\approx \;\frac{\rho \mathrm{Var}\{L\}-H\mathrm{Var}\{L\}}{{\bar{L}}^{2}}\;=\;\frac{\left(\rho -H)\mathrm{Var}\{L\right\}}{{\bar{L}}^{2}}\;=\;\frac{r\;\mathrm{Var}\{L\}}{{\bar{L}}^{2}}\;=\;O\left(r\right)\;=\;O\left({\bar{L}}^{-5/3}\right).$$

The bias coefficient shrinks at the same rate as the redundancy – not a second, independent achievement, but a direct consequence of the same design principle. Compare V2F: *ℓ* cannot track *L* at all, so Cov{L,l}=0 is permanent regardless of how the tree is built, and neither the $O(1/\bar{L})$ floor on *r* nor the resulting $C=\rho \mathrm{Var}\left\{L\right\}/{\bar{L}}^{2}$ can be improved by this mechanism.

For a binary Bernoulli(2/3) source, the explicit Bugeaud–Drmota–Szpankowski construction [20] (convergent denominator q=5) gives $\bar{L}=116.4$, ρ=0.9211 against H=0.9183 (r=0.0028), and Var{L}=4824; Equation (50) predicts C≈0.00097, close to the exact value C=0.000962 from Proposition 1 (Cov{L,l}=4430 against the predicted HVar{L}=4430). Table 3 confirms the resulting O(1/n) convergence numerically, using the exact moment formula, Equation (13), at every *n*:

The approximation is accurate to five decimal places by n=50, well before the asymptotic regime is actually reached.

## 5. Extension to Markov Sources

This section repeats the memoryless program of Section 3 with the i.i.d. assumption dropped: a boundary state is identified that renders successive phrases Markov rather than independent (Section 5.1); the scalar quantities $\mu_{j}\left(t\right)$ become matrices indexed by that state (Section 5.2); the mean formula of Theorem 3 becomes a matrix product (Section 5.4, validated against direct simulation); and the Laplace-method bias analysis of Section 3.5 becomes a matrix-eigenvalue perturbation, with the correction term now characterized by a Poisson equation rather than a plain derivative (Section 5.5, with a pointer to the underlying argument in Appendix B). The memoryless case reappears throughout as an exact special case, not a separate limit.

We now extend the source model to the Markov case. Specifically, in this section, $X_{1},X_{2},\ldots$ is assumed an irreducible, time-homogeneous, first-order Markov chain on the alphabet X of cardinality α, with transition probabilities $\mathrm{Pr}\{X_{k+1}=x^{\prime }|X_{k}=x\}$, and initial state $X_{0}$. The parsing tree, dictionary, and codeword assignment are exactly as before.

### 5.1. Source Model: The Boundary State $S_{i}$

Let $\Sigma_{i}=L\left(Y_{1}\right)+\cdots +L\left(Y_{i}\right)$ denote the total number of source symbols consumed through the *i*-th phrase (with $\Sigma_{0}\overset{\Delta }{=}0$, consistent with the $\Sigma_{n}$ of Section 2), so phrase $Y_{i}$ consists of the symbols $X_{\Sigma_{i-1}+1},\ldots ,X_{\Sigma_{i}}$. We define the state of the source at the *i*-th phrase boundary as the last symbol consumed before that boundary, that is,

(51) $$S_{i}\overset{\Delta }{=}X_{\Sigma_{i}},\;i=0,1,2,\ldots$$

and so $S_{0}=X_{0}$. Thus the state set is X itself, of size α; $S_{i-1}$ is the symbol in force when phrase $Y_{i}$ begins, and $S_{i}$ is the symbol reached when phrase $Y_{i}$ ends and the parser resets to the root of the parsing tree.

The initial state $S_{0}$ is taken to have the stationary distribution π of the phrase-boundary chain ${\left\{S_{i}\right\}}_{i\geq 0}$ just defined (shown to be a genuine Markov chain in Lemma 2 below)—a distribution generally different from the ordinary stationary distribution of ${\left\{X_{k}\right\}}_{k\geq 0}$ itself, since phrase boundaries sample states at variable, state-dependent intervals rather than at every symbol (Section 5.4 gives a numerical instance of this gap).

Since phrase $Y_{i}$ is parsed by running the parsing tree against the symbols $X_{\Sigma_{i-1}+1},X_{\Sigma_{i-1}+2},\ldots$ until a leaf is reached, the triple $\left(L\left(Y_{i}\right),l\left(Y_{i}\right),S_{i}\right)$ is a measurable function of $S_{i-1}=X_{\Sigma_{i-1}}$ together with the (as yet unconsumed) continuation of the chain from time $\Sigma_{i-1}$ onward. This lets the Markov property of ${\left\{X_{k}\right\}}_{k\geq 0}$ pass through the parsing recursion to the phrase level:

**Lemma** **2** (Markov property at phrase boundaries). *Conditioned on $S_{i-1}$, the triple $\left(L\left(Y_{i}\right),l\left(Y_{i}\right),S_{i}\right)$ is independent of $\left(S_{0},\ldots ,S_{i-2},Y_{1},\ldots ,Y_{i-1}\right)$, with conditional law depending on $S_{i-1}$ alone—and, by time-homogeneity, the same function of $S_{i-1}$ for every i, not merely free of the extra history at each fixed i. Consequently ${\left\{S_{i}\right\}}_{i\geq 0}$ is a time-homogeneous Markov chain on X.*

**Proof.**  $\left(L\left(Y_{i}\right),l\left(Y_{i}\right),S_{i}\right)$ is a function of $S_{i-1}=X_{\Sigma_{i-1}}$ and the continuation $\left(X_{\Sigma_{i-1}+1},X_{\Sigma_{i-1}+2},\ldots \right)$ alone, where $\Sigma_{i-1}$ is a stopping time of ${\left\{X_{k}\right\}}_{k\geq 0}$. By the strong Markov property, conditioned on $X_{\Sigma_{i-1}}=S_{i-1}$ this continuation is independent of the past $\left(X_{0},\ldots ,X_{\Sigma_{i-1}}\right)$—and so of

$$(S_{0},\ldots ,S_{i-2},Y_{1},\ldots ,Y_{i-1}),$$

functions of that past—with a law depending only on $S_{i-1}$, by time-homogeneity of the transition kernel.  □

This is what makes ${\left\{S_{i}\right\}}_{i\geq 0}$—not ${\left\{X_{k}\right\}}_{k\geq 0}$ itself—the Markov chain that matters for the moment analysis: the matrix quantities below are indexed by X and evolve one step per phrase rather than per symbol. The construction extends verbatim to a *k*-th order Markov chain or a hidden finite-state source, taking $S_{i}$ to be the last *k* symbols or the hidden state; we do not pursue this here.

### 5.2. Matrix-Valued Moment Generating Functions

For states $s,s^{\prime }\in X$, define the matrix-valued moment generating function

(52) $${\left[\mu_{j}\left(t\right)\right]}_{ss^{\prime }}:=E\left\{{\left[l\left(Y_{i}\right)\right]}^{j}e^{-tL(Y_{i})}1\left[S_{i}=s^{\prime }\right]|S_{i-1}=s\right\},\;j=0,1,2,\ldots ,\;\;t\geq 0,$$

for any phrase index i≥1 (by Lemma 2, this conditional law does not depend on *i*). These are α×α nonnegative matrices parameterized by *t*: $\mu_{j}\left(t\right)$ is the matrix analogue of the scalar quantity $\mu_{j}\left(t\right)$ of Section 2, and the case j=0 corresponds to the state transition matrix at phrase boundaries, ${\left[\mu_{0}\left(t\right)\right]}_{ss^{\prime }}=E\left\{e^{-tL(Y_{i})}1\left[S_{i}=s^{\prime }\right]|S_{i-1}=s\right\}$. At t=0, ${\left[\mu_{0}\left(0\right)\right]}_{ss^{\prime }}=\mathrm{Pr}\left\{S_{i}=s^{\prime }|S_{i-1}=s\right\}$, so $\mu_{0}\left(0\right)$ is row-stochastic.

We assume throughout that $\mu_{0}\left(0\right)$—the phrase-boundary chain’s own transition matrix, as distinct from the transition matrix of ${\left\{X_{k}\right\}}_{k\geq 0}$ itself—is irreducible and aperiodic (equivalently, primitive); this is a separate condition from the irreducibility of ${\left\{X_{k}\right\}}_{k\geq 0}$ assumed in Section 5 (a tree can route every path ending in a given state through a first symbol unreachable in one step from another state, so the two irreducibilities do not simply hand each other over), and it holds for every example in this paper. Aperiodicity is needed, not just irreducibility: an irreducible but periodic chain can have several eigenvalues tied for maximum modulus (e.g., the period-2 chain $\left(\begin{array}{cc} 0 & 1\\ 1 & 0 \end{array}\right)$ has eigenvalues 1 and −1, both of modulus 1), which would break the spectral-gap argument used in Section 5.5 and Appendix B to isolate the dominant eigenvalue’s contribution; primitivity is exactly what rules this out (by the Perron–Frobenius theorem for primitive matrices, the Perron eigenvalue is then strictly greater in modulus than every other eigenvalue).

This is a simple sufficient condition, easily checked without appeal to Perron–Frobenius theory directly: if ${\left\{X_{k}\right\}}_{k\geq 0}$ has a fully positive one-step transition matrix (i.e., $\mathrm{Pr}\{X_{k+1}=x^{\prime }|X_{k}=x\}>0$ for every $x,x^{\prime }\in X$), then $\mu_{0}\left(0\right)$ has strictly positive entries throughout, and is therefore automatically both irreducible and aperiodic. Indeed, full positivity means any specific finite symbol sequence has positive probability of occurring next, from any current state; since the parsing tree is finite, every leaf corresponds to some such sequence, so every leaf—and hence every ending state—is reachable with positive probability from every starting state in a single phrase. This condition is considerably stronger than needed (it fails, for instance, whenever some transition is structurally forbidden), but it covers most sources encountered in practice and requires no computation beyond inspecting the source’s own transition matrix.

Under this assumption, let π denote the unique stationary distribution of $\mu_{0}\left(0\right)$—a row vector, consistent with its left-multiplying matrices throughout (as in $\pi \mu_{0}\left(0\right)$ just below)—satisfying $\pi \mu_{0}\left(0\right)=\pi$ and $\sum_{s}\pi_{s}=1$. For a state-dependent single-phrase quantity $G(Y_{i})$ (such as the phrase length $L(Y_{i})$, the codeword length $l(Y_{i})$, or their product), we write $E_{\pi }\left\{G\left(Y_{i}\right)\right\}\overset{\Delta }{=}\sum_{s\in X}\pi_{s}E\left\{G\left(Y_{i}\right)|S_{i-1}=s\right\}$ for its expectation under the stationary regime; for G=L, this plays the role of $\bar{L}$ of Section 2 in the Markov case, and is the a.s. limit $\Sigma_{n}/n\to E_{\pi }\left\{L\left(Y_{i}\right)\right\}$ by the ergodic theorem for irreducible finite-state Markov chains.

### 5.3. The First Moment Formula for the Markov Case

**Theorem** **5.** *If the initial state $S_{0}$ has the stationary distribution π of the phrase-boundary chain, then*

(53) $$E\left\{R_{n}\right\}=\int_{0}^{\infty }\pi \left[\sum_{i=1}^{n}\mu_{0}{\left(t\right)}^{i-1}\mu_{1}\left(t\right)\mu_{0}{\left(t\right)}^{n-i}\right]1dt,$$

*where 1 is the all-ones column vector and π is understood to be a row vector.*

**Proof.**  As in the proof of Theorem 3, $E\left\{R_{n}\right\}=\int_{0}^{\infty }E\left\{\Lambda_{n}e^{-t\Sigma_{n}}\right\}dt$. Expanding $\Lambda_{n}e^{-t\Sigma_{n}}=\sum_{i=1}^{n}l\left(Y_{i}\right)e^{-t(L\left(Y_{1}\right)+\cdots +L\left(Y_{n}\right))}$,

(54) $$E\left\{\Lambda_{n}e^{-t\Sigma_{n}}\right\}=\sum_{i=1}^{n}E\left\{l\left(Y_{i}\right)e^{-tL(Y_{i})}\prod_{j\neq i}e^{-tL(Y_{j})}\right\}.$$

For a given *i*, define $h_{j}\overset{\Delta }{=}e^{-tL(Y_{j})}$ for j≠i and $h_{i}\overset{\Delta }{=}l\left(Y_{i}\right)e^{-tL(Y_{i})}$, so the *i*-th term of Equation (54) is $E\{\prod_{j=1}^{n}h_{j}\}$. For j=1,…,n+1, define

(55) $$\phi_{j}\left(s\right)\overset{\Delta }{=}E\left\{\prod_{k=j}^{n}h_{k}|S_{j-1}=s\right\},\;s\in X$$

where it should be understood that the product is empty when j=n+1, so $\phi_{n+1}\equiv 1$. This is well-defined as a function of *s* alone—a conditional expectation given a random variable is, by definition, always some function of that variable—with no appeal to the Markov property yet. What the Markov property buys us is that these functions also satisfy the backward recursion

(56) $$\phi_{j}\left(s\right)=E\left\{h_{j}\;\phi_{j+1}\left(S_{j}\right)|S_{j-1}=s\right\},\;j=n,n-1,\ldots ,1,$$

i.e., that $\phi_{j}$ can be built from $\phi_{j+1}$ one phrase at a time, rather than recomputing a fresh conditional expectation over k=j,…,n from scratch at every step. We prove Equation (56) via the following stronger claim, by induction on *j* decreasing from n+1 to 1:

(57) $$E\left\{\prod_{k=j}^{n}h_{k}\;|\;S_{0},\ldots ,S_{j-1},Y_{1},\ldots ,Y_{j-1}\right\}=\phi_{j}\left(S_{j-1}\right),$$

where conditioning on the entire history through phrase j−1, rather than on $S_{j-1}$ alone as in Equation (55), changes nothing. The case j=n+1 is immediate: both sides equal 1, the product on the left being empty and $\phi_{n+1}\equiv 1$ by definition.

For the inductive step from j+1 to *j* (assuming Equation (57) at j+1, prove it at *j*), condition on $\left(S_{0},\ldots ,S_{j},Y_{1},\ldots ,Y_{j}\right)$ and use Equation (57) at j+1:

(58) $$E\left\{\prod_{k=j}^{n}h_{k}|S_{0},\ldots ,S_{j-1},Y_{1},\ldots ,Y_{j-1}\right\}=E\left\{h_{j}\;\phi_{j+1}\left(S_{j}\right)|S_{0},\ldots ,S_{j-1},Y_{1},\ldots ,Y_{j-1}\right\}.$$

By Lemma 2 applied to phrase *j*, $h_{j}\;\phi_{j+1}\left(S_{j}\right)$—a function of $\left(L\left(Y_{j}\right),l\left(Y_{j}\right),S_{j}\right)$—is independent of $\left(S_{0},\ldots ,S_{j-2},Y_{1},\ldots ,Y_{j-1}\right)$ given $S_{j-1}$, so the right-hand side reduces to $E\{h_{j}\;\phi_{j+1}\left(S_{j}\right)|S_{j-1}\}$, a function of $S_{j-1}$ alone—giving Equation (57) at *j*, and, comparing this same computation against definition Equation (55) of $\phi_{j}$, exactly the recursion Equation (56). Only the one-step property of Lemma 2 is used, once per step of the induction; the recursion is what accumulates it, phrase by phrase, into Equation (57).

Taking j=1 in Equation (57) gives $E\left\{\prod_{k=1}^{n}h_{k}|S_{0}=s\right\}=\phi_{1}\left(s\right)$—automatic from Equation (55) itself at j=1, and a useful consistency check; averaging over $S_{0}∼\pi$ gives $E\left\{\prod_{j=1}^{n}h_{j}\right\}=\sum_{s}\pi_{s}\phi_{1}\left(s\right)$. Unwinding the recursion Equation (56) and recognizing ${\left[\mu_{0}\left(t\right)\right]}_{ss^{\prime }}$, ${\left[\mu_{1}\left(t\right)\right]}_{ss^{\prime }}$ from Equation (52) at each step (the matrix at step *j* is $\mu_{1}\left(t\right)$ if j=i and $\mu_{0}\left(t\right)$ otherwise),

(59) $${\vec{\phi }}_{1}={\left[\mu_{0}\left(t\right)\right]}^{i-1}\mu_{1}\left(t\right){\left[\mu_{0}\left(t\right)\right]}^{n-i}\;1,$$

where ${\vec{\phi }}_{1}$ is the column vector of values $\phi_{1}\left(s\right)$, s∈X. Hence, the *i*-th term of Equation (54) equals $\pi {\left[\mu_{0}\left(t\right)\right]}^{i-1}\mu_{1}\left(t\right){\left[\mu_{0}\left(t\right)\right]}^{n-i}1$; summing over i=1,…,n and integrating over *t* gives Equation (53).  □

### 5.4. Worked Example and Validation

We use the binary Markov source of Savari and Gallager [6]: the state is the last bit emitted, and the self-transition probability (probability of emitting the same bit as the last one) is *q*. This source has two states {0,1}, with transition probabilities $\mathrm{Pr}[S_{i}=s|S_{i-1}=s]=q$ and $\mathrm{Pr}[S_{i}=1-s|S_{i-1}=s]=1-q$.

We use the parsing dictionary {00,01,1} (the same as in Section 4) and assign fixed-length codewords l≡2 (a V2F code). The three possible phrases and their contributions to $\mu_{0}\left(t\right)$ are:

- Phrase 1 (one symbol equal to 1): Emitted from state 0 with probability 1−q (cross-transition) and from state 1 with probability *q* (self-transition), driving the source to state 1, with L=1.
- Phrase 00 (two 0s): Reached via symbol 0 then another 0; probability from state 0 is $q\cdot q=q^{2}$ and from state 1 is (1−q)·q=q(1−q), driving the source to state 0, with L=2.
- Phrase 01: From state 0 via 0 (prob. *q*) then 1 (prob. 1−q), probability q(1−q); from state 1 via 0 (prob. 1−q) then 1 (prob. 1−q), probability ${\left(1-q\right)}^{2}$. Drives source to state 1, L=2.

Collecting these contributions into the matrix $\mu_{0}\left(t\right)$ (rows indexed by starting state, columns by ending state),

(60) $$\mu_{0}\left(t\right)=\left(\begin{array}{cc} q^{2}e^{-2t} & \left(1-q\right)e^{-t}+q\left(1-q\right)e^{-2t}\\ q\left(1-q\right)e^{-2t} & qe^{-t}+{\left(1-q\right)}^{2}e^{-2t} \end{array}\right).$$

Since l≡2, $\mu_{1}\left(t\right)=2\mu_{0}\left(t\right)$ and Equation (53) simplifies to $E\left\{R_{n}\right\}=2n\int_{0}^{\infty }\pi \mu_{0}{\left(t\right)}^{n}1\;dt$.

**Example** **1.** *Take q=0.99 (a highly persistent source). The stationary distribution of parsing-point states is found by solving $\pi \mu_{0}\left(0\right)=\pi$, $\pi_{0}+\pi_{1}=1$, giving π=(0.332,0.668)—visibly different from the ordinary stationary distribution of the symbol-level chain ${\left(X_{k}\right)}_{k\geq 0}$ itself, which is (1/2,1/2) for every q by the symmetry of its transition probabilities under 0↔1; this is the numerical instance, promised in Section 5.1, of the general fact that phrase-boundary sampling need not preserve a chain’s own stationary law. Evaluating Equation (53) by numerical quadrature at n=10 phrases gives $E\{R_{10}\}=1.6503$. A Monte Carlo simulation of 2,000,000 independent realizations of the Markov parsing process gives $\hat{E\{R_{10}\}}=1.6506\pm 0.0003$, in agreement within one standard error. Evaluating Equation (53) at increasing n (using the t=u/n substitution and full eigendecomposition of $\mu_{0}\left(t\right)$ for numerical stability at large n) gives the finite-n convergence reported in Table 4.*

*The sequence decreases monotonically to the ergodic limit $\rho =E_{\pi }\left\{l\right\}/E_{\pi }\left\{L\right\}=2/\left(\pi_{0}\cdot 1.99+\pi_{1}\cdot 1.01\right)=2/1.336=1.497$. As a cross-check of the formula, write Λ(t) for the dominant (Perron) eigenvalue of $\mu_{0}\left(t\right)$—guaranteed simple and positive by the irreducibility of $\mu_{0}\left(0\right)$, and strictly greater in modulus than every other eigenvalue by the additional aperiodicity assumed in Section 5.2—with v(t) its corresponding right eigenvector and u(t) its corresponding left eigenvector, normalized so u(t)v(t)≡1. At t=0, since $\mu_{0}\left(0\right)$ is row-stochastic, Λ(0)=1 with v(0)=1 (as $\mu_{0}\left(0\right)1=1$) and u(0)=π (as $\pi \mu_{0}\left(0\right)=\pi$, and indeed π1=1, consistent with the normalization). Differentiating the eigenvalue equation $\mu_{0}\left(t\right)v\left(t\right)=\Lambda \left(t\right)v\left(t\right)$ at t=0 and left-multiplying by u(0)=π,*

(61) $$\pi \mu_{0}^{\prime }\left(0\right)v\left(0\right)+\pi \mu_{0}\left(0\right)v^{\prime }\left(0\right)=\Lambda^{\prime }\left(0\right)\;\pi v\left(0\right)+\Lambda \left(0\right)\;\pi v^{\prime }\left(0\right);$$

*since $\pi \mu_{0}\left(0\right)=\pi =\Lambda \left(0\right)\pi$, the second term on each side is the same, $\pi v^{\prime }\left(0\right)$, and cancels, leaving $\Lambda^{\prime }\left(0\right)=\pi \mu_{0}^{\prime }\left(0\right)1$ (using v(0)=1). Since ${\left[\mu_{0}\left(t\right)1\right]}_{s}=\sum_{s^{\prime }}{\left[\mu_{0}\left(t\right)\right]}_{ss^{\prime }}=E\left\{e^{-tL(Y_{i})}|S_{i-1}=s\right\}$ (the indicator $1[S_{i}=s^{\prime }]$ summed over $s^{\prime }$ is just 1), differentiating at t=0 gives $\mu_{0}^{\prime }\left(0\right)1=-g$ with $g_{s}:=E\left\{L\left(Y_{i}\right)|S_{i-1}=s\right\}$, so $\Lambda^{\prime }\left(0\right)=-\pi g=-\sum_{s}\pi_{s}g_{s}=-E_{\pi }\left\{L\right\}$ by the definition of $E_{\pi }\left\{L\right\}$ in Section 5.2 (this same g reappears as the reward vector of the Poisson equation in Section 5.5 below). Numerically, $\Lambda^{\prime }\left(0\right)=-2\left(q+1\right)/\left(2q+1\right)=-1.336$ (obtained directly by differentiating the characteristic polynomial of $\mu_{0}\left(t\right)$ at t=0); indeed, $-\Lambda^{\prime }\left(0\right)=1.336=E_{\pi }\left\{L\right\}$, confirming the general formula just derived.*

### 5.5. Asymptotic Bias for Markov Sources

The large-*n* asymptotic analysis of Equation (53) proceeds as in Section 3.5: the substitution t=u/n stabilizes the numerical evaluation, and the resulting bias coefficient $n(E\left\{R_{n}\right\}-\rho )$ converges to a finite limit as n→∞. This is governed by the dominant eigenvalue Λ(t) of $\mu_{0}\left(t\right)$ and the reason is that, for large *n*, ${\left[\mu_{0}\left(t\right)\right]}^{n}$ itself is governed by ${\left[\Lambda \left(t\right)\right]}^{n}$ (times a bounded projector term, standard for a matrix with a strictly dominant eigenvalue)—the matrix analogue of the scalar ${\left[\mu_{0}\left(t\right)\right]}^{n}$ of the memoryless case, which trivially equals itself raised to the *n*-th power. So extracting the O(1/n) term from Equation (53) requires a Taylor series expansion of Λ(t) around t=0 to the same order that Section 3.5 needed for the scalar quantity $f\left(t\right)=-\ln\mu_{0}\left(t\right)$. For Example 1, the results are displayed in Table 5.

A closed-form expression for the bias coefficient requires going one order further than the eigenvalue derivative $\Lambda^{\prime }\left(0\right)=-E_{\pi }\left\{L\right\}$ already computed (Example 1)—exactly as the memoryless case of Section 3.5 needed not just $f^{\prime }\left(0\right)$ but also $f^{\prime \prime }\left(0\right)$. For a matrix eigenvalue problem, unlike the scalar case, getting the second-order term $\Lambda^{\prime \prime }\left(0\right)$ requires first finding the first-order correction to Λ(t)’s corresponding eigenvector—a standard fact of matrix perturbation theory (the same mechanism as second-order energy shifts needing first-order wavefunction corrections in perturbation theory more generally)—and this correction is what the Poisson equation below solves for.

Specifically, consider the expansion $\mu_{0}\left(t\right)=P+t\mu_{0}^{\prime }\left(0\right)+O\left(t^{2}\right)$ (with $P\overset{\Delta }{=}\mu_{0}\left(0\right)$), Λ(t)’s corresponding right eigenvector as $v\left(t\right)=1+t\;e+O\left(t^{2}\right)$ (since P1=1, this right eigenvector at t=0 is 1, and *e* is its unknown first-order correction), and the eigenvalue itself as $\Lambda \left(t\right)=1-\bar{L}t+O\left(t^{2}\right)$, where $\bar{L}:=E_{\pi }\left\{L\right\}$. Substituting into the eigenvalue equation $\mu_{0}\left(t\right)v\left(t\right)=\Lambda \left(t\right)v\left(t\right)$ and matching the coefficients of *t* on both sides gives

(62) $$\left(P-I\right)e=-\bar{L}1-\mu_{0}^{\prime }\left(0\right)1.$$

Recall from Example 1 that ${\left[\mu_{0}\left(t\right)1\right]}_{s}=E\left\{e^{-tL(Y_{i})}|S_{i-1}=s\right\}$, so $\mu_{0}^{\prime }\left(0\right)1=-g$ with $g_{s}\overset{\Delta }{=}E\left\{L\left(Y_{i}\right)|S_{i-1}=s\right\}$ the expected phrase length given starting state *s*. Substituting into Equation (62),

(63) $$\left(P-I\right)e=g-\bar{L}1,\;i.e.,\;\left(I-P\right)e=\bar{L}1-g.$$

Since (I−P)1=0, this equation only determines *e* up to an additive multiple of 1; writing $w\overset{\Delta }{=}-e$ and fixing this freedom by the normalization πw=0 turns it into exactly the Poisson equation

(64) $$\left(I-P\right)w=g-E_{\pi }\left\{L\right\}\cdot 1,\;\pi w=0,$$

(this name and normalization are standard in Markov reward theory [26], where (I−P)w=h with πh=0 is the equation for the relative reward *w* associated with a per-state reward whose π-average has already been subtracted off). Here *w*—the relative-value vector—is precisely the relative-reward vector of Savari and Gallager [6], computed there for the reward “self-information of a phrase” under dictionary-size asymptotics; here, the reward is “phrase length *L*” and the asymptotics are in phrase count.

This Poisson-equation correction is in fact the whole of what is needed: a second, completely analogous Poisson equation for the reward *ℓ*, combined with a joint eigenvalue perturbation in both the reward variable and *t*, yields the bias coefficient *C* in closed form for a general Markov V2V code—not merely the V2F sub-case tabulated above (writing $\Lambda_{\theta t}$ for the resulting mixed second derivative, and $\Lambda^{\prime \prime }\left(0\right)$ for the second derivative of the already-familiar Λ(t) of Example 1):

**Proposition** **3** (Markov bias coefficient)**.** *For a Markov V2V code whose phrase-boundary chain is irreducible and aperiodic (Section 5.2), with stationary distribution π, phrase-length mean $\bar{L}\overset{\Delta }{=}E_{\pi }\left\{L\right\}$, codeword-length mean $\bar{l}\overset{\Delta }{=}E_{\pi }\left\{l\right\}$ (so $\rho =\bar{l}/\bar{L}$), and $\bar{c}\overset{\Delta }{=}E_{\pi }\left\{Ll\right\}$,*

(65) $$\lim_{n\to \infty }n\left(E\{R_{n}\}-\rho \right)=C=\frac{\bar{l}\;\Lambda^{\prime \prime }\left(0\right)+\bar{L}\;\Lambda_{\theta t}}{{\bar{L}}^{3}},$$

*where*

(66) $$\Lambda^{\prime \prime }\left(0\right)=\pi \;\mu_{0}^{\prime \prime }\left(0\right)\;1-2\;\pi \;\mu_{0}^{\prime }\left(0\right)\;w,\;\Lambda_{\theta t}=-\bar{c}-\pi \;\mu_{1}\left(0\right)\;w+\pi \;\mu_{0}^{\prime }\left(0\right)\;w_{l},$$

*w solves Equation (64) and $w_{l}$ solves the analogous equation $\left(I-P\right)w_{l}=g_{l}-\bar{l}\;1$, $\pi w_{l}=0$, with $g_{l}\left(s\right)\overset{\Delta }{=}E\left\{l\left(Y_{i}\right)|S_{i-1}=s\right\}$.*

We omit the full coefficient-by-coefficient derivation here, for reasons of length and because it follows, essentially step for step, the same pattern already carried out in full for the memoryless case (Section 3.5): Taylor-expand an eigenvalue via its eigenvector, solve a Poisson equation via a Fredholm-type compatibility condition, and assemble. The complete derivation is given in the extended version of this paper [27], and the resulting formula is additionally verified numerically to 10+ significant figures against two different Markov V2V codes in Appendix B below.

**Proof** **sketch.** The argument extends the single-variable eigenvalue perturbation above ($\Lambda^{\prime }\left(0\right)=-\bar{L}$, via the Poisson equation for *w*) to two variables: a second, completely analogous Poisson equation for the reward *ℓ* produces $w_{l}$, and a joint perturbation of the dominant eigenvalue in both the reward variable and *t* together produces $\Lambda^{\prime \prime }\left(0\right)$ and the new mixed term $\Lambda_{\theta t}$. Appendix B gives the two-line pointer to this argument; the formula is verified numerically to 10+ significant figures against direct high-precision computation of the exact formula Equation (53) at large *n*, on two different Markov V2V codes, and collapses exactly to Proposition 1 in the memoryless limit.  □

**Example** **2** (Closed form for Example 1). *Applying Proposition 3 to the V2F code of Example 1 (l≡2, so $\mu_{1}\left(t\right)=2\mu_{0}\left(t\right)$ and $\bar{c}=2\bar{L}$) gives, after simplification,*

(67) $$C\left(q\right)=\frac{q(2q^{2}-q+1)}{2\left(1-q\right){\left(1+q\right)}^{3}}.$$

*At q=0.99, C(0.99)=12.3753, matching the numerical target of the table above (validated independently to 10 significant figures against direct high-precision computation of Equation (53) at n up to $10^{9}$).*

**Example** **3** (A genuine V2V code under the same Markov source)**.** *Proposition 3 applies equally when ℓ is itself random and state-dependent. Take the same Savari–Gallager source and dictionary {00,01,1}, now equipped with the Huffman codeword assignment of Section 4, (l(00),l(01),l(1))=(1,2,2)—a genuine V2V code, since both L and ℓ vary across phrases. Here ℓ depends only on the destination state (l=1 when the parser returns to the root via “00”, l=2 otherwise), which gives ρ(q)=(3q+2)/[2(q+1)] and, applying Proposition 3,*

(68) $$C\left(q\right)=\frac{q(4q^{2}+q+1)}{4\left(1-q\right){\left(1+q\right)}^{3}}.$$

*At q=0.99: ρ=1.2487 and C(0.99)=18.5623, again validated to 10 significant figures against direct high-precision computation of Equation (53), and against an independent check using a second codeword assignment for which ℓ does not depend only on the destination state.*

**Figure 2:**
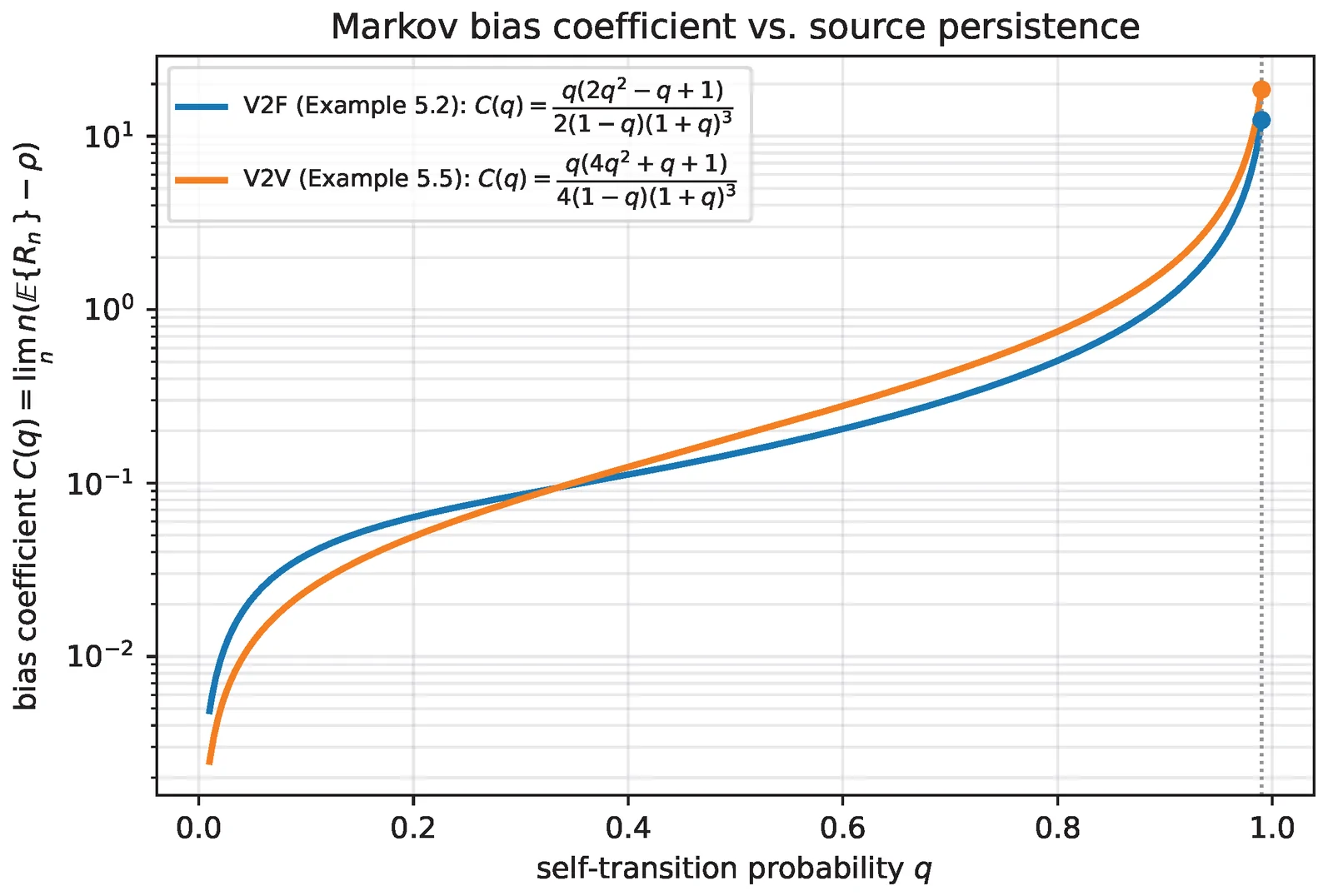
The Markov bias coefficient C(q) of Proposition 3, for the V2F code of Example 2 and the V2V code of Example 3, as the self-transition probability *q* ranges over (0,1). Markers indicate the q=0.99 values reported in the text.

Figure 2 plots both closed forms Equations (67) and (68) over the full range q∈(0,1), rather than at the single value q=0.99 tabulated above. Both bias coefficients diverge as q→1—a highly persistent source mixes slowly, and the Poisson-equation solutions $w,w_{l}$ that drive $\Lambda^{\prime \prime }\left(0\right)$ and $\Lambda_{\theta t}$ grow correspondingly large—and, notably, the two curves cross at exactly q=1/3 (as follows directly from equating Equations (67) and (68)): for weakly persistent sources the V2F code has the larger finite-*n* bias, while for strongly persistent sources (including the q=0.99 case tabulated in both examples) the genuine V2V code’s bias is larger, mirroring the memoryless-case tension already noted in Section 4 between optimising a code for ρ and optimising it for *C*.

## 6. Conclusions

The exact formula Equation (19) gives, for any fixed V2V code applied to a DMS, exact formulas for all integer moments of the realized compression ratio $R_{n}$ at every finite *n*: mean, variance, and skewness are computable by one-dimensional numerical quadrature, from which an Edgeworth approximation to the CDF is obtained. The Laplace method applied to Equation (19) recovers the classical ratio-estimator bias and variance asymptotics algebraically, by a different route than the delta method (Section 3.5), and provides a design guideline (maximize Cov{L,l} for fastest convergence) backed by a precise, finite-*n* formula.

The extension of Equation (53) to Markov sources replaces scalar quantities by matrix-valued ones and is validated both against direct simulation and against the correct ergodic limit; the O(1/n) bias coefficient for this Markov case is likewise obtained in closed form (Proposition 3, Appendix B, via a joint eigenvalue perturbation that reduces exactly to Proposition 1 in the memoryless limit).

Independently of the moment analysis, the observation that a V2V code is an instance of a finite-state encoder lets us directly apply the generalized Kraft inequality of [2]: via Corollary 1, this gives an explicit, *m*-independent lower bound on the compression ratio in terms of the dictionary parameters *M*, α, and $l_{\max}$, with an O(1/m) correction term that improves on the Ziv–Lempel bound whose analogous constant grows linearly in *m*. The structural analysis of Section 4 decomposes the bias coefficient *C* into a Var{L} term and a Cov{L,l} term, each of which vanishes identically for one of F2V and V2F; applied to the Khodak code, the same decomposition shows that its improved $O({\bar{L}}^{-5/3})$ redundancy and its correspondingly smaller bias coefficient are two faces of the same design principle rather than independent achievements.

## Figures and Tables

**Table 1:** Notation used across more than one section of the paper, with pointers to where each symbol is first defined. Purely local symbols, introduced and used within a single proof, are omitted.

Symbol	Meaning	First Defined
*Source and code structure*		
X, α	source alphabet, its cardinality	Section 2
D, *M*	dictionary (leaf set), its size	Section 2
$Y_{i}$, $L(Y_{i})$, $l(Y_{i})$	*i*-th phrase, its length, its codeword length	Section 2
H(X)	source symbol entropy	Section 2
H(Y)	source phrase entropy	Section 2
$\bar{L},\bar{l}$	mean phrase length, mean codeword length	Section 2
ρ	asymptotic compression rate, $\bar{l}/\bar{L}$	Section 2
$\Lambda_{n},\Sigma_{n},R_{n}$	total codeword length, total symbols, $R_{n}=\Lambda_{n}/\Sigma_{n}$	Section 2
K, spr(K)	Kraft matrix, its spectral radius	Section 2
*Memoryless moment analysis (Section 3)*		
$\mu_{j}\left(t\right)$	single-phrase quantity $E\{{\left[l\left(Y\right)\right]}^{j}e^{-tL(Y)}\}$	Section 2
f(t)	$-\ln\mu_{0}\left(t\right)$, negative log of the MGF of −L(Y)	Section 3.5
$\kappa_{2}$, $c_{lL}$	Var{L(Y)}, E{l(Y)L(Y)}	Section 3.5
*C*	O(1/n) bias coefficient, $E\{R_{n}\}\to \rho +C/n$	Section 3.5
$\gamma_{1}$	skewness of $R_{n}$	Section 3.2
*Markov extension (Section 5)*		
$X_{k}$, $S_{i}$	underlying symbol chain, phrase-boundary state $X_{\Sigma_{i}}$	Section 5.1
$\mu_{j}\left(t\right)$	matrix analogue of $\mu_{j}\left(t\right)$, indexed by X	Section 5.2
π	stationary distribution of $\mu_{0}\left(0\right)$ (row vector)	Section 5.2
$E_{\pi }\left\{Q\right\}$	$\sum_{s}\pi_{s}E\left\{Q\left(Y_{i}\right)|S_{i-1}=s\right\}$	Section 5.2
Λ(t)	dominant (Perron) eigenvalue of $\mu_{0}\left(t\right)$	Section 5.4
$\bar{c}$	$E_{\pi }\left\{Ll\right\}$ (and $\bar{L},\bar{l}$ reused for $E_{\pi }\left\{L\right\},E_{\pi }\left\{l\right\}$)	Section 5.5
$g_{s}$	$E\{L\left(Y_{i}\right)|S_{i-1}=s\}$	Section 5.4
$g_{l}\left(s\right)$	$E\{l\left(Y_{i}\right)|S_{i-1}=s\}$	Section 5.5
*w*, $w_{l}$	Poisson-equation solutions for the rewards *L*, *ℓ*	Section 5.5

**Table 2:** Absolute CDF approximation error at n=50, against empirical CDF from 2,000,000 Monte Carlo trials.

*z*	*x*	CLT Error	Edgeworth Error
−2.0	0.6434	0.00773	0.00004
−0.5	0.7287	0.01881	0.00614
−0.0	0.7571	0.01972	0.00058
−0.5	0.7855	0.01111	0.00155
−2.0	0.8708	0.00574	0.00203

**Table 3:** Exact vs. first-order asymptotic $E\{R_{n}\}$ for the explicit Khodak code of [20], illustrating the O(1/n) convergence rate of Proposition 1.

n	Exact $E\{R_{n}\}$	Asymp. ρ+C/n	Error (Approx.−Exact)
1	0.91994	0.92206	+0.00213
2	0.92269	0.92158	−0.00111
5	0.92144	0.92129	−0.00015
10	0.92123	0.92120	−0.000033
20	0.92116	0.92115	−0.0000082
50	0.92112	0.92112	−0.0000013
100	0.92111	0.92111	≈0
*∞*	ρ=0.92110	ρ=0.92110	0

**Table 4:** Finite-*n* convergence of $E\{R_{n}\}$ to the ergodic limit *ρ*, for the Markov worked example of Example 1.

n	5	10	30	(∞)
$E\{R_{n}\}$	1.657	1.650	1.627	ρ=1.497

**Table 5:** Numerical convergence of the bias coefficient $n(E\left\{R_{n}\right\}-\rho )$ to its limit, for Example 1.

*n*	2000	10,000	50,000	(n→∞)
$E\{R_{n}\}$	1.504	1.499	1.498	1.497
$n(E\left\{R_{n}\right\}-\rho )$	12.2	12.3	12.4	≈12.4

## Data Availability

No new data were created or analyzed in this study. Data sharing is not applicable to this article.

## Appendix A. Proof of the Boundary Laplace Lemma

This appendix proves Lemma 1, the boundary case of Laplace’s method used in Section 3.5, in its general form (arbitrary k,m≥1).

**Proof** **of Lemma 1.** Substitute t=u/n; then $t^{k-1}dt=u^{k-1}du/n^{k}$, and

(A1) $$\frac{n!}{(n-m)!}\int_{0}^{\infty }t^{k-1}h\left(t\right)e^{-(n-m)f(t)}dt=\frac{n!}{\left(n-m\right)!\;n^{k}}\int_{0}^{\infty }u^{k-1}h\left(u/n\right)e^{-(n-m)f(u/n)}du.$$

Now,

(A2) $$\frac{n!}{(n-m)!}=n\left(n-1\right)\cdots \left(n-m+1\right)=n^{m}\left[1-\frac{m(m-1)}{2n}+O\left(\frac{1}{n^{2}}\right)\right].$$

Now, consider the Taylor series expansion of −f around t=0: $-f\left(t\right)=-at+\frac{b}{2}t^{2}+O\left(t^{3}\right)$ as t→0, so with *u* fixed and n→∞,

(A3) $$-\left(n-m\right)f\left(u/n\right)=\left(n-m\right)\left[-\frac{au}{n}+\frac{bu^{2}}{2n^{2}}\right]+O\left(\frac{1}{n^{2}}\right)=-au+\frac{u}{n}\left(ma+\frac{b}{2}u\right)+O\left(\frac{1}{n^{2}}\right),$$

whence

(A4) $$e^{-(n-m)f(u/n)}=e^{-au}\left[1+\frac{u}{n}\left(ma+\frac{b}{2}u\right)+O\left(\frac{1}{n^{2}}\right)\right].$$

Expanding *h* as a Taylor series, we have $h\left(u/n\right)=h_{0}+\frac{h_{1}u}{n}+O\left(1/n^{2}\right)$, and multiplying the two expansions, we obtain

(A5) $$u^{k-1}h\left(u/n\right)e^{-(n-m)f(u/n)}=e^{-au}\left[u^{k-1}h_{0}+\frac{1}{n}\left(mah_{0}u^{k}+\frac{b}{2}h_{0}u^{k+1}+h_{1}u^{k}\right)\right]+O\left(n^{-2}\right).$$

Integrating term by term using the identity $\int_{0}^{\infty }u^{k-1+j}e^{-au}du=\left(k-1+j\right)!/a^{k+j}$,

(A6) $$\int_{0}^{\infty }u^{k-1}h\left(u/n\right)e^{-(n-m)f(u/n)}du=\frac{\left(k-1\right)!h_{0}}{a^{k}}+\frac{1}{n}\left(\frac{mh_{0}k!}{a^{k}}+\frac{bh_{0}\left(k+1\right)!}{2a^{k+2}}+\frac{h_{1}k!}{a^{k+1}}\right)+O\left(\frac{1}{n^{2}}\right).$$

Finally, multiplying by $\frac{n!}{\left(n-m\right)!n^{k}}=n^{m-k}\left(1-\frac{m(m-1)}{2n}+O\left(1/n^{2}\right)\right)$ and collecting the two leading powers of *n*, the O(1/n) coefficient becomes

(A7) $$\frac{mh_{0}k!}{a^{k}}+\frac{bh_{0}\left(k+1\right)!}{2a^{k+2}}+\frac{h_{1}k!}{a^{k+1}}-\frac{m(m-1)}{2}\cdot \frac{\left(k-1\right)!h_{0}}{a^{k}}=\frac{(k-1)!}{2a^{k+2}}\left(a^{2}h_{0}m\left(2k-m+1\right)+2ah_{1}k+bh_{0}k\left(k+1\right)\right),$$

using 2mk−m(m−1)=m(2k−m+1) to combine the $h_{0}$-terms. This is exactly Equation (33).  □

## Appendix B. Closed-Form Markov Bias Coefficient

The derivation extends the eigenvalue-perturbation argument of Section 3.5 to a joint matrix-valued moment generating function Ψ(θ,t), indexed by the phrase-boundary states, whose (0,t)-slice is $\mu_{0}\left(t\right)$ and whose θ-derivative at θ=0 is $\mu_{1}\left(t\right)$. Differentiating the eigenvalue equation for Ψ’s dominant (Perron) eigenvalue Λ(θ,t) in each direction, and using the standard compatibility condition for the resulting singular linear system at each order, gives the first-order coefficients $\Lambda_{t}\left(0,0\right)=-\bar{L}$ and $\Lambda_{\theta }\left(0,0\right)=\bar{l}$ together with the two Poisson-equation solutions $w,w_{l}$ already stated in Section 5.5; differentiating once more, using *w* and $w_{l}$, yields $\Lambda^{\prime \prime }\left(0\right)$ and the mixed term $\Lambda_{\theta t}$ quoted in Proposition 3. The resulting formula was verified numerically to 10+ significant figures against direct high-precision computation of the exact formula Equation (53) at large *n*, on two different Markov V2V codes built on the source of Example 1 (one with *ℓ* depending only on the destination state, one without that special structure), and collapses exactly to Proposition 1 in the memoryless limit.
